# Mimicry in the Bite: Shared Sequences Between *Aedes aegypti* Salivary Proteins and Human Proteins

**DOI:** 10.3390/proteomes13040056

**Published:** 2025-11-03

**Authors:** Andrea Arévalo-Cortés, Daniel Rodriguez-Pinto

**Affiliations:** 1 Carrera de Medicina, Facultad de la Salud Humana, Universidad Nacional de Loja, Loja 110103, Ecuador; andrea.arevalo@unl.edu.ec; 2 Carrera de Medicina, Departamento de Ciencias de la Salud, Facultad de Ciencias de la Salud, Universidad Técnica Particular de Loja, Loja 110108, Ecuador

**Keywords:** molecular mimicry, autoimmunity, *Aedes aegypti*, dengue, Zika, yellow fever

## Abstract

Background: Molecular mimicry contributes to the development of unwanted responses to self-antigens. Autoimmune phenomena have been observed in diseases caused by *Aedes aegypti*-transmitted arboviruses, but the occurrence of mimicry between salivary and human proteins has been unexplored. Methods: We used bioinformatic tools to determine if peptides from *Aedes aegypti* salivary proteins were present in the human proteome. We further characterized the potential of shared sequences to induce immunity by analyzing their predicted binding to MHC molecules and their occurrence in peptides from the Immune Epitope Database (IEDB). Results: We analyzed 9513 octapeptides from 29 *Aedes aegypti* salivary proteins against the human proteome and found 47 peptides identical to sequences from 52 human proteins, ranging in length from 8 to 18 amino acids. We found 302 matches of peptides predicted to bind with high affinity to MHC-I and MHC-II alleles associated with autoimmune diseases, and 14 human peptides containing shared sequences with *Aedes aegypti* salivary proteins validated as immunogenic in the IEDB. Conclusions: These results support the existence of molecular mimicry between *Aedes aegypti* salivary proteins and human antigens and provide a framework for studies to determine its contribution to responses directed to self-antigens in the context of arboviral infections.

## 1. Introduction

*Aedes aegypti* is a mosquito vector responsible for transmitting several arboviruses that cause significant morbidity globally, including dengue virus (DENV), Zika virus (ZIKV), yellow fever virus, and Chikungunya virus (CHIKV) [1,2]. The diseases caused by these viruses are complex and include severe presentations, the pathophysiology of which is poorly understood [3,4,5,6]. In dengue, severe disease is characterized by plasma leakage and bleeding caused by platelet, coagulation, and endothelial cell dysfunction, which are a consequence of an excessive inflammatory response [7,8,9]. Autoimmune phenomena have been proposed as a contributing factor due to the finding in affected patients of DENV-specific antibodies that cross react with endothelial cells, platelets, coagulation factors, and plasminogen that can alter their normal function [7,10,11,12,13,14,15,16]. DENV infection has also been reported as the trigger for the development of autoimmune pathology, including lupus and neurological disorders [17,18,19,20]. Autoimmunity has also been implicated in neurological complications of ZIKV infection, including Guillain-Barré syndrome, transverse myelitis, and dysautonomia [21,22,23]. These observations are strengthened by several case reports that link Guillain-Barré syndrome to DENV [24,25], CHIKV [26], and ZIKV [27,28,29,30]. Furthermore, West Nile virus, another vector-borne virus, has also been linked to Guillain-Barré syndrome [31,32] and autoimmune encephalitis [33].

The genesis of autoimmunity remains obscure, but it is accepted that in most cases several genetic and environmental factors must concur to cause the breakdown of tolerance to self-antigens [34,35,36]. Molecular mimicry, the existence of very similar structures in two different organisms capable of igniting a cross-reactive immune response, has been recognized as an important factor in several autoimmune diseases [37,38,39]. Abundant evidence exists for viral and bacterial antigens cross-reacting with autoantigens recognized in human diseases, but only in a few entities, such as rheumatic fever [40] and Guillain-Barré syndrome [41], is molecular mimicry believed to be the main pathological mechanism. In most cases, its contribution has not been clearly established, but it is believed to be an important trigger. The occurrence of identical amino acid sequences that lead to epitope sharing between microorganisms and humans has been extensively documented for numerous viruses [42,43,44,45], bacteria [46,47,48], and parasites [49,50], as well as their capacity to ignite both humoral and cellular immune responses [47,51,52,53]. In severe dengue, mimicry between DENV proteins such as NS1, E, and prM and human antigens expressed in endothelial cells and platelets, as well as coagulation factors, has been documented and proposed as the origin of autoimmune responses that contribute to pathogenesis [13,14,54,55,56,57,58,59]. Likewise, significant molecular mimicry exists between the ZIKV proteome and human proteins related to neurological structures affected in Zika infection [60,61].

During arbovirus infection, salivary proteins from the mosquito vector enter the human body and immediately are found in an immunogenic environment promoted by activation of innate immune receptors by viral pathogen-associated molecular patterns (PAMPs) [62,63,64] and effects derived from the mosquito bite, which include skin trauma, salivary factors, and microbiota [65,66,67]. Thus, the potential for them to include shared epitopes with human proteins is relevant, as molecular mimicry may contribute to the autoimmune phenomena associated with *Aedes aegypti*-transmitted diseases. Although many roles for *Aedes aegypti* salivary proteins have been described, including modulation of the immune response [68,69,70] and altering of the coagulation and vascular systems [71,72,73,74], no studies have addressed the issue of mimicry with human proteins. However, antibodies specific for *Aedes* salivary proteins have been detected in humans [75,76,77,78], thus confirming their immunogenic potential. Interestingly, molecular mimicry between human desmoglein 1 and *Lutzomyia longipalpis* and *Phlebotomus papatasi* salivary proteins has been proposed as a mechanism for pemphigus foliaceus [79].

Here, we used bioinformatic methods to determine if protein sequences of eight amino acids or more are shared between *Aedes aegypti* salivary proteins and human proteins. We found 47 shared sequences between 21 *Aedes aegypti* proteins and 60 human proteins, representing linear epitopes with the potential of activating autoreactive lymphocytes. We further determined the existence of peptides containing these sequences that bind MHC molecules with high affinity and to have been tested for immune reactivity.

## 2. Materials and Methods

### 2.1. Determination of Occurrence of Identical Peptide Sequences in Proteins from Aedes aegypti Saliva and Humans

Sequences from 29 proteins expressed in *Aedes aegypti* saliva [80,81,82,83] were retrieved from the National Center for Biotechnology Information (NCBI) protein database (Table 1). The Peptide Library Design Tool from GenScript (https://www.genscript.com/peptide_screening_tools.html (accessed on 3 September 2025)) was used to generate an octapeptide library with an overlap of seven amino acids. The length of eight amino acids was chosen because this is the shortest length that can represent a linear peptide capable of activating T cells, as MHC-I molecules bind peptides from 8 to 15 amino acids in length [84], while MHC-II molecules bind peptides from 11 to 30 amino acids in length [85]. Identical sequences of at least eight amino acids were searched in each of the peptides generated using the NCBI Protein Basic Local Alignment Search Tool (BLAST) (https://blast.ncbi.nlm.nih.gov/Blast.cgi?PROGRAM=blastp&PAGE_TYPE=BlastSearch&LINK_LOC=blasthome (accessed on 3 September 2025)) by entering the sequence for each peptide and running the search for non-redundant protein sequences against the human proteome (*Homo sapiens* (PARIS) (taxid: 9606)) using the blastp algorithm. Search parameters were adjusted automatically by the search tool to search for a short input sequence. Results were filtered to include only sequences with 100% identity. The octapeptide library with a shift of one amino acid (overlap of seven amino acids) covered the whole sequence of each protein with the maximum amount of octapeptides, ensuring that no identical sequence of at least eight amino acids could be absent from the results. To determine if the identical sequences found were longer than eight amino acids, peptides including the flanking amino acids of each of these sequences were aligned with the corresponding matched human protein using blastp. The E value (number of alignment scores that would be expected to be found by chance with an alignment score equal or better than the observed alignment) was determined for each identified identical sequence within the matched human proteins. When the results showed multiple entries for the same protein (including isoforms) in the human proteome only one was chosen for analysis and the others were registered in an Excel file (Supplementary File S1). Sequences that matched hypothetical or uncharacterized human proteins, as well as variable regions of antigen receptors, were not considered in the results. Post-translational modifications affecting amino acids from each shared sequence were searched for in the UniProt (https://www.uniprot.org/ (accessed on 3 September 2025)) entry of each *Aedes aegypti* salivary protein and human protein. Each bioinformatic analysis described was run one time and the relevant results were registered in an Excel file (Supplementary File S1).

### 2.2. Prediction of Peptide Binding Affinity for MHC Molecules

Binding affinity of peptides containing sequences shared between *Aedes aegypti* salivary proteins and human proteins was predicted using T Cell Prediction—Class I and T Cell Prediction—Class II tools available in the Immune Epitope Database (IEDB) website (https://nextgen-tools.iedb.org/ (accessed on 10 September 2025)). For MHC-II predictions, the identified shared sequences from the salivary proteins were extended on both sides until a peptide of 18 to 19 amino acids was reached. These sequences were entered into the Class II tool with the following parameters: peptide length: 11–16 amino acids; peptide shift length: 1; MHC alleles: a panel of 30 MHC-II alleles associated with autoimmune diseases was selected (Table 2). For DQ genes, alleles are available in the prediction tool only as DQA1/DQB1 combinations. For this reason, the 10 combinations available for each DQA1 allele in Table 2 were selected and a total of 50 HLA-DQA1/HLA-DQB1 alleles were analyzed; prediction model: NetMHCIIpan 4.1 EL. Peptide–MHC predicted interactions with a median binding percentile < 1 were considered of high affinity. For each sequence that had at least one peptide with high binding affinity in the *Aedes* protein, the same process was carried out for the matching sequence of the human protein. For an *Aedes* peptide and a human peptide to be considered a match for high-affinity MHC binding the following conditions were required: both had a median binding percentile < 1; their length was equal, they were aligned for the shared sequence, and they had at least seven continuous amino acids from the shared sequence. Each bioinformatic analysis described was run one time and the relevant results were registered in an Excel file (Supplementary File S2).

For MHC-I predictions, the identified shared sequences from the salivary proteins were extended on both sides until a peptide of 10 to 11 amino acids was reached. These sequences were entered into the Class I tool with the following parameters: peptide length: 8–9 amino acids; MHC alleles: a panel of 22 MHC-I alleles associated with autoimmune diseases was selected (Table 3); prediction model: NetMHCpan 4.1 EL. Peptide–MHC predicted interactions with a median binding percentile < 1 were considered of high affinity. For each sequence that had at least one peptide with high binding affinity in the *Aedes* protein, the same process was carried out for the matching sequence of the human protein. For an *Aedes* peptide and a human peptide to be considered a match for high-affinity MHC binding the following conditions were required: both had a median binding percentile < 1 (meaning that any random peptide would have a 99% probability of having a lower binding affinity to that particular MHC allele); their length was equal, they were aligned for the shared sequence, and they had at least seven continuous amino acids from the shared sequence. Each bioinformatic analysis described was run one time and the relevant results were registered in an Excel file (Supplementary File S3).

### 2.3. Determination of the Presence of Sequences Shared Between Aedes aegypti Salivary Proteins and Human Proteins in Validated Peptides from the Immune Epitope Database

Sequences shared between *Aedes Aegypti* salivary proteins and human proteins were entered into the Immune Epitope Database (IEDB) search tool (https://www.iedb.org/ (accessed on 15 September 2025)) set to BLAST—70% and restricted to positive assays. Peptides resulting from this search that originated from the same human protein that shared a sequence with the corresponding *Aedes aegypti* salivary protein were chosen. To identify matched peptides between *Aedes aegypti* and human proteins, sequences were aligned at the start of the shared sequence present in the IEDB human peptide, and the sequence of the *Aedes aegypti* protein was extended until a peptide of the same size was obtained. A match was defined by the following conditions: at least 85% identity between the peptides and at least seven continuous amino acids from the identical shared sequence in both peptides. Each bioinformatic analysis described was run one time.

## 3. Results

### 3.1. Occurrence of Identical Peptide Sequences in Aedes aegypti Salivary Proteins and Human Proteins

A total of 9513 octapeptides obtained from 29 *Aedes aegypti* salivary proteins were probed to find identical sequences of at least eight amino acids in the human proteome. The complete results can be found in Supplementary File S1 and are summarized in Table 4 and Table 5. As shown in Table 4, a total of 47 peptides from 21 salivary proteins had 60 identical matches in 52 different human proteins. The length of the matching peptides ranged from 8 to 18 amino acids, with 32 out of the 47 (68.1%) being octamers. Lymphotoxin β receptor inhibitor (LTRIN), aldehyde dehydrogenase, ficolin, angiopoietin, and α-glucosidase were the salivary proteins with most matches, accounting for 28 (59.6%) of the peptides and 40 (66.7%) of the 60 matches found in human proteins.

The 47 shared peptides with the 60 matching human proteins are shown in Table 5. For the *Aedes aegypti* salivary protein LTRIN, which is the one with the most matching sequences, eight of the nine peptides are found in two regions of the protein that contain a repetition of glutamine residues (YQQQQQQQPQ, amino acids 32–41; and PQQQQQQHQQP, amino acids 59–69). These peptides include one decamer, two nonamers, and five octamers that match sequences in 17 distinct human proteins. Seven of the identified peptides were matched between the *Aedes aegypti* protein and its human homolog, including three enzymes (amylase, adenosine deaminase, and aldehyde dehydrogenase) and the innate immune receptor ficolin. Only 2 of the 60 matches (3.3%) contained amino acids that were the site of post-translational modifications in human proteins: sites for lysine ubiquitination in aldehyde dehydrogenase 3 and for threonine phosphorylation in phosphatidylinositol (PI)-binding clathrin assembly protein (Supplementary File S1). No post-translational modifications were found in the shared sequences in *Aedes aegypti* salivary proteins. In seven instances (five *Aedes aegypti* salivary proteins and two human proteins) the shared sequences were totally or partially part of the signal sequence of a secreted protein. This does not invalidate their potential for immunogenicity, as signal peptides have been found to be presented in the context of MHC molecules [115].

### 3.2. High-Affinity Binding of Peptides Containing Shared Sequences Between Aedes aegypti Salivary Proteins and Human Proteins to MHC-II Molecules

**Figure 1:**
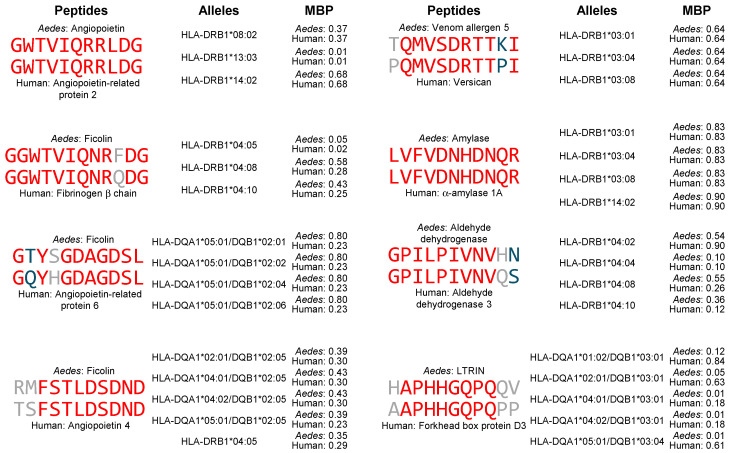
Representative examples of matching peptides from *Aedes aegypti* salivary proteins and human proteins that contain shared sequences and bind with high affinity to MHC-II alleles associated with autoimmune diseases. Peptides are aligned to the shared sequence. Identical amino acids are shown in red and similar amino acids are shown in blue. Amino acids shown in gray are neither identical nor similar. MBP: mean binding percentile.

T cell activation requires the ligation of the T cell receptor (TCR) by a complex formed between a peptide from the immune response-inducing protein and an MHC molecule [116]. The very high polymorphism of the genes that encode human MHC molecules ensures that virtually any peptide can be presented in this fashion at the population level [117]. However, the high-affinity binding of peptides to MHC molecules is correlated to higher efficiency for T cell activation [118,119]. For this reason, we sought to determine if the prediction of peptide binding to MHC-II molecules indicated that the identified shared sequences between *Aedes aegypti* salivary proteins and human proteins were included in peptides that bind with very high affinity to a panel of 72 MHC-II alleles that have been associated with autoimmunity (Table 2) following the strategy described in the Materials and Methods Section. The results of this analysis are found in Supplementary File S2 and are summarized in Table 6 and Table 7, while Figure 1 shows the representative examples of high affinity binding matching peptides.

We found 69 peptides from *Aedes aegypti* salivary proteins that bound with high affinity (<1 median binding percentile) to at least one of the MHC-II alleles associated with autoimmunity and had a matching peptide in at least one human protein. As shown in Table 6, the matched peptides were from six *Aedes aegypti* proteins and nine human proteins. A total of 259 matches were found that had high-affinity binding to 46 different MHC-II alleles. Angiopoietin is the *Aedes aegypti* salivary protein with most matches, which includes 23 different *Aedes aegypti* peptides matched to peptides from three different human proteins; these matching peptides bound to three MHC-II alleles with high affinity. Likewise, 14 peptides from *Aedes aegypti* ficolin matched peptides from three human proteins that bind 25 MHC-II alleles for a total of 67 matches. LTRIN, aldehyde dehydrogenase, amylase and venom allergen-5 were the other *Aedes aegypti* proteins for which matches were found, all for peptides from one human protein each. Since the length of peptides that bind MHC-II is greater than most of the shared peptides identified in our analysis, our strategy included extending these peptides and thus the resulting matches have different sequences. Thus, we only found 19 out of 259 (7.3%) identical matches, 12 of them for the longest sequence (ALVFVDNHDNQRGHGAGG from amylase). However, as shown in Table 5 and illustrated in eight representative examples in Figure 1, 144 of 259 (55.6%) matching peptides had at least 80% identity.

The frequency of matches for the 46 alleles had a wide distribution (range 1 to 58 matches), with the highest frequency observed for the three alleles that mainly bound peptides from angiopoietin (Table 7). In fact, the 11 alleles with the highest frequencies (5 or more matches) accounted for 67.2% of the matches (174 of 259), while the other 35 alleles had four or less matches each and together accounted for 32.8% of the matches (85 of 259). This analysis allows us to conclude that the shared sequences we identified can be presented as peptide–MHC-II complexes in a potentially efficient manner by binding with high affinity, although most of the instances are concentrated in a relatively small proportion of the elements probed (4 mosquito proteins and 11 MHC-II alleles).

### 3.3. High-Affinity Binding of Peptides Containing Shared Sequences Between Aedes aegypti Salivary Proteins and Human Proteins to MHC-I Molecules

**Figure 2:**
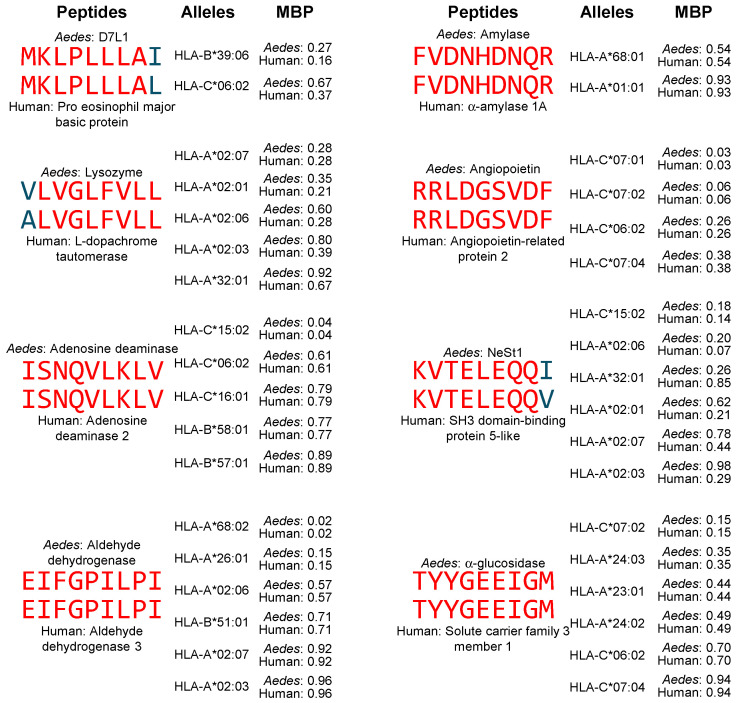
Representative examples of matching peptides from *Aedes aegypti* salivary proteins and human proteins that contain shared sequences and bind with high affinity to MHC-I alleles associated with autoimmune diseases. Peptides are aligned to the shared sequence. Identical amino acids are shown in red and similar amino acids are shown in blue. MBP: mean binding percentile.

CD8^+^ T cell responses are also capable of contributing to autoimmune pathology through molecular mimicry [52,120]. For this reason, we predicted high-affinity binding of the peptides shared by *Aedes aegypti* salivary proteins and human proteins for 22 MHC-I alleles associated with autoimmunity (Table 3). The results of this analysis are found in Supplementary File S3 and are summarized in Table 8 and Table 9, while Figure 2 shows representative examples of high-affinity-binding matching peptides.

A total of 19 peptides from 10 *Aedes aegypti* salivary proteins bound with high affinity to MHC-I alleles associated with autoimmunity, matching peptides from 14 human proteins. The highest frequency of matches was found for aldehyde dehydrogenase, which had ten matches involving nine different alleles, while ficolin, angiopoietin, and α-glucosidase had six matches each involving six alleles each (Table 8). Since the length of peptides that bind MHC-I molecules is short, we limited our analysis for octamers and nonamers and extended the octamers by one amino acid. Thus, all matches were either identical or had only one non-identical amino acid, as illustrated in Figure 2 and shown in the total identical matches in Table 8 (22 of 43, 51.2%).

Matches with high-affinity binding were found for 19 of 22 (86.4%) investigated alleles, albeit none of them had more than four matches. As shown in Table 9, HLA-A*02:07, HLA-C*06:02, HLA-C*15:02, and HLA-C*16:01 were the alleles with the most matches. In conclusion, this analysis indicates that peptides from Ae. *aegypti* salivary proteins that have shared sequences with human proteins can also be presented in the context of MHC-I molecules in a highly stable and immunogenic form, albeit with a lower frequency than that found for MHC-II peptide binding.

### 3.4. Peptides in the Immune Epitope Database Containing Sequences Shared Between Aedes aegypti Salivary Proteins and Human Proteins

**Figure 3:**
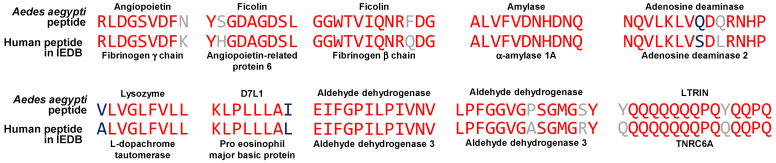
Matching peptides from IEDB and *Aedes aegypti* salivary proteins. Identical amino acid residues are shown in red and similar amino acid residues in blue. Amino acids shown in gray are neither identical nor similar. The sequences RLDGSVDF and GGWTVIQNR had more than one matching peptide from the corresponding human protein; only the one with highest identity is shown.

Next, we searched for peptides in the IEDB that originated from the previously identified human proteins that contain sequences shared with *Aedes aegypti* salivary proteins. We found that 10 of the sequences from 10 different human proteins were included in 14 IEDB-validated peptides that matched an *Aedes aegypti* salivary protein peptide (matching peptides had ≥85% identity and at least seven continuous amino acids from the shared sequence). Figure 3 shows ten of these peptides aligned with each *Aedes aegypti* matching peptide. Two of the matching peptides were identical and two others had 100% similarity. Table 10 shows the validated characteristics for each peptide. Interestingly, four peptides that matched with sequences from *Aedes aegypti* ficolin, D7L1, and LTRIN were validated in the context of autoimmune pathology, highlighting the potential for cross-reactive immune responses.

## 4. Discussion

The fact that the etiology of most autoimmune diseases remains obscure, despite all the accumulated knowledge regarding the immune response, points to multiple significant factors concurring and interacting in a complex fashion [34,35,36]. In this context, the description of new mechanisms for self-antigens to be targeted is a relevant contribution to the advancement of understanding of autoimmunity. The possibility of receiving protein sequences encountered on human proteins in an immunogenic form after a mosquito bite has barely been explored, and thus the bioinformatic approach taken in this present study to prove the existence of shared sequences between *Aedes aegypti* salivary proteins and human antigens represents the first step toward establishing a novel manner for molecular mimicry to inform autoimmune pathology. By searching for the occurrence of octapeptides derived from the *Aedes aegypti* salivary proteins in the human proteome, we found 47 shared peptides of 8 to 18 amino acids in length. Our analysis of MHC-binding and validation in the IEDB indicates that several of these linear sequences have the potential of generating ligands for both CD4^+^ and CD8^+^ T cells leading to effector mechanisms that would target the human tissues that express the human antigen due to cross-reactivity.

The shared peptides described in this analysis were found in 52 human proteins that encompass a variety of locations and functions, as summarized in Table 11. Self-antigens targeted in autoimmunity are not restricted to any category [138,139], and therefore it is possible for each of these proteins to contribute to an autoimmune response. For instance, intracellular antigens that are not normally exposed to the immune system can trigger an immune response if liberated because of inadequate clearance of apoptotic cells or tissue damage due to inflammation [140,141]. In this case, the presence of cross-reactive T cells previously primed by the contact with saliva could thus induce damaging inflammation. As shown in Table 11, we found 30 intracellular proteins, 20 of which had ubiquitous distribution.

Another important aspect of autoimmune phenomena in the context of infectious diseases is their contribution to exacerbating or inhibiting the host response. Both directions can support the pathogenesis of severe disease in *Aedes aegypti*-transmitted arboviral infections, as inhibition can result in increased viral loads and excessive inflammation can damage tissues [193,194]. Any autoimmune response that exacerbates inflammation is a potential contributor to the hyperinflammatory state that characterizes severe presentations of Aedes aegypti-transmitted infections, leading to cytokine storm, vascular leakage, and organ dysfunction [9,195,196], and in some cases neural invasion due to increased permeability of the blood–brain barrier [197,198]. Thus, an immune response to several membrane and secreted proteins that have a shared sequence with *Aedes aegypti* salivary proteins and participate in inflammation, immune recognition, wound healing, hemostasis, and cell adhesion (Table 11) could affect the host response.

High-affinity binding of peptides to MHC molecules has been associated with the effectiveness of the immune response [118] and is a requisite for MHC–peptide complex stability, which has been shown to be a determinant for immunogenicity [119,199,200]. Our analysis found 259 matching peptides in *Aedes aegypti* salivary proteins and human proteins that bound to MHC-II alleles associated with autoimmune diseases with very high affinity (mean binding percentile lower than 1) and 43 such matches for MHC-I. Only three *Aedes aegypti* salivary proteins included peptides with matches for both types of MHC molecules: angiopoietin, ficolin, and aldehyde dehydrogenase. Together, they represented 75.2% (227 of 302) of the matches, indicating that the shared peptides found in these proteins can be stably presented in alleles associated with a variety of autoimmune conditions. Since MHC alleles are considered an important genetic factor in the development of autoimmunity [87,88,89,99,102], there is a potential for these proteins to contribute to the generation of a pathogenic autoimmune response in susceptible individuals.

One of the limitations of this study is that the analysis used peptides from the reference sequence for each *Aedes aegypti* salivary protein against the reference human proteome and thus does not account for all possible proteoforms. The existence of proteoforms can undoubtedly affect immunogenicity as fine structural details can alter T cell activation [201]; accordingly, post-translational modifications have been proven to be a critical factor in the genesis of autoimmunity [202]. We determined that most of the shared sequences we found are not affected by post-translational modifications, but due to their high complexity, assessing the impact of the existence of proteoforms in the potential of shared sequences to ignite autoimmune responses is not possible with this analysis.

The future directions required to validate molecular mimicry between *Aedes aegypti* salivary proteins and human proteins as triggers of autoimmune pathology encompass several steps: (1) demonstration of cross-reactive T cells and antibodies; (2) generation of an autoimmune response in an animal model of the infection after administering the antigens containing the shared sequences; and 3) establishing an epidemiological relationship between mosquito bites and autoimmune pathologies [38,51]. Thus, immunological and epidemiological studies are needed, along with the development of animal models that reflect severe presentations and chronic complications of *Aedes aegypti*-transmitted infections.

## 5. Conclusions

Although *Aedes aegypti* salivary proteins have been characterized as antibody-inducing antigens [75,76,77,78] and allergens [203,204,205], their immunogenic potential in the context of autoimmunity has not been explored. Our bioinformatic analysis shows the sharing of identical peptides between *Aedes aegypti* salivary proteins and human proteins, as well as their potential to be immunogenic by binding with high affinity to MHC molecules. This information can direct further studies to evaluate the impact of autoimmune responses to vector saliva in *Aedes aegypti*-transmitted infections and can also aid in enhancing the security of salivary protein-based vaccines [206,207,208].

## Figures and Tables

**Table 1:** *Aedes aegypti* salivary proteins analyzed for matching identical sequences in the human proteome.

Protein	GenBank Accession Number	Length (Amino Acids)	Octapeptides Probed
AaVA-1	A0A1S4EWW7.1	255	248
Adenosine deaminase	XP_021698106.1	530	523
Aegyptin	O01949.2	273	266
AeMOPE-1	Q8T9V8.1	95	88
AgBR1	ABF18180.1	439	432
Aldehyde dehydrogenase	AGI96742.1	494	487
α-glucosidase	P13080.1	579	572
Amylase	AAB60935.1	737	730
Angiopoietin	ABF18152.1	354	347
Apyrase	P50635.2	562	555
CLIPA3	A0A6I8TBG6.1	472	465
C-type lectin	ABF18475.1	163	156
D7L1	P18153.2	321	314
D7L2	Q0IF93.1	332	325
Ficolin	JAN95606.1	283	276
LTRIN	J9HGJ1.1	210	203
Lysozyme	AAU09087.1	148	141
Mucin 2	ABF18030.1	281	274
Mucin 3	JAN95076.1	292	285
Mucin 4	JAN95079.1	574	567
Mucin 6	JAN95078.1	130	123
NeSt1	Q17F11.1	316	309
PE-binding protein	ABF18501.1	224	217
Proline-rich mucin	ABF18067.1	100	93
Purine hydrolase	AAL76010.1	338	331
Serpin	ABF18509.1	417	410
SG34	A0A1S4F550.1	312	305
Sialokinin	P42634.2	85	78
Venom allergen 5	NP_001395334.1	200	193
	Total	9516	9513

AaVA: *Aedes aegypti* venom allergen; AeMOPE: *Aedes*-specific modulatory peptide; AgBR: *Aedes aegypti* bacteria-responsive protein; CLIP: cytoplasmic linker protein; LTRIN: lymphotoxin β receptor inhibitor; NeSt: neutrophil-stimulating factor; PE: phosphatidylethanolamine.

**Table 2:** MHC-II alleles associated with autoimmune diseases analyzed for binding to *Aedes aegypti* and human peptides with matching sequences.

MHC-II Allele		Associated Autoimmune Disease	References
HLA-DQA1*	01:02	SLE; MS	[86,87]
	02:01	Psoriasis; T1D	[88]
	04:01	T1D; SLE	[87,89]
	04:02	SLE	[88]
	05:01	T1D; GD	[89,90,91,92]
HLA-DQB1*	02:01	T1D; celiac disease; SLE; MG	[87,90,91,93,94,95]
	03:02	RA; T1D; PR; UC; pemphigus	[87,90,91,94,96]
	03:03	MG; psoriasis	[88,97]
HLA-DRB1*	01:01	RA; AIH	[98,99,100,101]
	01:02	RA	[102]
	03:01	SLE; T1D; MS; RA; AIH; SS; MG	[86,89,90,91,93,94,95,99,100,103,104,105]
	03:04	GD; MS	[92,106]
	03:07	RA	[88]
	03:08	RA	[88]
	04:01	RA; PR; MS; T1D; AIH	[87,94,98,99,100,101,103]
	04:02	Pemphigus	[96]
	04:04	T1D; PR; MS; RA	[87,90,98,99,103]
	04:05	T1D; RA	[90,98,101]
	04:08	RA	[98]
	04:10	RA	[101]
	07:01	AIH; RA; CD	[89,100,102]
	08:02	SLE	[88]
	09:01	RA; T1D; MG	[89,97,99]
	10:01	RA	[99,101]
	13:03	MS	[103]
	14:01	ITP; MS; GD	[103,107,108]
	14:02	RA	[98,99]
	15:01	SLE; thyroid autoimmune disease; MS; RA	[86,87,89,102,103]
	15:02	SLE; UC	[89,109]
	16:02	SLE	[88]

SLE: systemic lupus erythematosus; RA: rheumatoid arthritis; T1D: type 1 diabetes; MS: multiple sclerosis; UC: ulcerative colitis; CD: Crohn’s Disease; PR: polymyalgia rheumatica; AIH: autoimmune hepatitis; MG: myasthenia gravis; SS: Sjögren’s syndrome; ITP: immune thrombocytopenic purpura; GD: Graves’ disease.

**Table 3:** MHC-I alleles associated with autoimmune diseases analyzed for binding to *Aedes aegypti* and human peptides with matching sequences.

MHC-I Allele		Associated Autoimmune Disease	References
HLA-A*	02:07	Psoriasis; MG	[97,110]
	24:02	T1D; MG	[97,102,111,112]
	24:03	T1D	[102,112]
HLA-B*	07:02	T1D	[111]
	08:01	MG	[95]
	18:01	T1D	[102,111]
	35:02	T1D	[111]
	37:01	MS; GD	[103,108]
	39:06	T1D	[102,111]
	44:03	T1D	[111]
	46:01	MG	[97]
HLA-C*	01:02	MG; T1D	[97,112]
	03:02	T1D	[112]
	03:03	T1D	[111]
	06:02	Psoriasis	[110,113,114]
	07:01	Psoriasis; GD	[102,110]
	07:02	SLE; psoriasis; GD	[86,102,110]
	07:04	Psoriasis; GD	[102,110]
	08:02	GD; T1D	[88,108]
	12:02	Psoriasis	[110]
	15:02	MS	[102]
	16:01	Psoriasis	[114]

SLE: Systemic lupus erythematosus; T1D: type 1 diabetes; MS: multiple sclerosis; GD: Graves’ disease; MG: myasthenia gravis.

**Table 4:** Summary of identical sequences matches found in *Aedes aegypti* salivary proteins and human proteins.

Protein		Peptides with Match							Matches in Human Proteome
	18-mer	13-mer	12-mer	11-mer	10-mer	9-mer	8-mer	Total	
LTRIN	0	0	0	0	1	2	6	9	18
Aldehyde dehydrogenase	0	0	1	1	0	1	4	7	7
Ficolin	0	0	0	0	0	2	3	5	5
Angiopoietin	0	1	0	0	0	0	3	4	7
α-glucosidase	0	0	0	0	1	1	1	3	3
AgBR1	0	0	0	0	0	0	2	2	2
Lysozyme	0	0	0	0	0	1	1	2	2
Mucin 3	0	0	0	0	0	0	2	2	2
NeSt1	0	0	0	0	0	0	1	1	1
Adenosine deaminase	0	0	0	0	1	0	0	1	1
Aegyptin	0	0	0	0	0	0	1	1	2
Amylase	1	0	0	0	0	0	0	1	1
Apyrase	0	0	0	0	0	0	1	1	1
D7L1	0	0	0	0	0	0	1	1	1
Mucin 4	0	0	0	0	0	0	1	1	1
Mucin 6	0	0	0	0	1	0	0	1	1
Proline-rich mucin	0	0	0	0	0	0	1	1	1
Purine hydrolase	0	0	0	0	0	0	1	1	1
Serpin	0	0	0	0	0	0	1	1	1
SG34	0	0	0	0	0	0	1	1	1
Venom allergen 5	0	0	0	0	0	0	1	1	1
Total	1	1	1	1	4	7	32	47	60

AgBR: *Aedes aegypti* bacteria-responsive protein; LTRIN: lymphotoxin β receptor inhibitor; NeSt: neutrophil-stimulating factor.

**Table 5:** Peptides that were identical matches in *Aedes aegypti* salivary proteins and human proteins.

*Aedes aegypti* Protein	Peptide	Length (Amino Acids)	Human Proteins
Amylase Angiopoietin	ALVFVDNHDNQRGHGAGG	18	α-amylase 1A
	GWTVIQRRLDGSV	13	Angiopoietin-related protein 2
	GWTVIQRR	8	Angiopoietin-related protein 4
			Angiopoietin-related protein 6
			Fibrinogen-like 1
	GEYWLGLE	8	Angiopoietin-related protein 6
			Angiopoietin-related protein 2
	RLDGSVDF	8	Fibrinogen γ chain
Aldehyde dehydrogenase	EIFGPILPIVNV	12	Aldehyde dehydrogenase 3
	TLELGGKSPCY	11	Aldehyde dehydrogenase 3
	GQTCIAPDY	9	Aldehyde dehydrogenase 3
	LELGGKSP	8	Cytosolic 10-formyltetrahydrofolate dehydrogenase
	KFLQEARS	8	Tyrosine-protein kinase
	SLPFGGVG	8	Aldehyde dehydrogenase 3
	LASSRYPP	8	INO80 complex subunit E
Adenosine deaminase	PISNQVLKLV	10	Adenosine deaminase
Mucin 6	STPSSSNSTS	10	PI-binding clathrin assembly protein
LTRIN	QQQQQQHQQP	10	GRB10-interacting GYF protein 2
	QQQQQQHQQ	9	Mixed-lineage leukemia 2
			Mediator of RNA polymerase II transcription
			Histone-lysine N-methyltransferase
	QQQQQQQPQ	9	Zinc finger homeobox protein 3
			TNRC6A
	QQQQQQHQ	8	CHD-1
			Ataxin 1
			Tumor protein 63
	PQQQQQQH	8	Transcriptional activator MN1
	QQQQQQPQ	8	DNA polymerase subunit γ-1
			MIDEAS
	QQQQQQQP	8	Ataxin 2
			Huntingtin
			Nuclear receptor corepressor 2
			Tensin-1
	YQQQQQQQ	8	AP2-associated protein kinase 1 isoform 4
			Zinc finger RNA-binding protein
	APHHGQPQ	8	Forkhead box protein D3
α-glucosidase	KDSDGDGIGD	10	Cartilage oligomeric matrix protein
	TYYGEEIGM	9	SLC3A1
	YPRSFKDS	8	SLC3A1
Lysozyme	LVGLFVLLA	9	L-dopachrome tautomerase
Ficolin	GGWTVIQNR	9	Fibrinogen β chain
	FSTLDSDND	9	Angiopoietin-4
	DGEFWLGL	8	Angiopoietin-related protein 3
	GDAGDSLS	8	Angiopoietin-related protein 6
	SNLNGLYL	8	Ficolin
Aegyptin	EEENEGEE	8	PAT1 homolog 2
			Tubulin α-8 chain
AgBR1	VSANNATT	8	RPTP-α
	FDGLDLAW	8	Chitinase-3-like protein 1
Mucin 3	LLIGAVLA	8	Dopamine receptor D4
	ELDSSDEE	8	DC-STAMP domain-containing protein 2
D7L1	MKLPLLLA	8	Pro eosinophil major basic protein
NeSt1	KVTELEQQ	8	SH3 domain-binding protein 5-like
Apyrase	KLTVGKRK	8	Zinc finger MYM-type protein 4
Lysozyme	CSLAKALL	8	Rho GEF 37
Mucin 4	DDDGDDFD	8	Nucleophosmin
Proline-rich mucin	SLLLILSI	8	NADH dehydrogenase
Purine hydrolase	DQDGGGDD	8	N-lysine methyltransferase SETD6
Serpin	DVLSKLKE	8	SCAPER
SG34	EVQLLRES	8	ER to nucleus signaling 1
Venom allergen 5	QMVSDRTT	8	Versican

AgBR: *Aedes aegypti* bacteria-responsive protein; LTRIN: lymphotoxin β receptor inhibitor; NeSt: neutrophil-stimulating factor; CHD-1: chromodomain-helicase-DNA-binding protein-1; MIDEAS: mitotic deacetylase-associated SANT domain protein; GEF: guanine nucleotide exchange factor; TNRC6A: trinucleotide repeat containing adaptor 6A; SCAPER: S phase cyclin A-associated protein in the endoplasmic reticulum; PI: phosphatidylinositol; RPTP: receptor-type tyrosine-protein phosphatase; SLC3A1: solute carrier family 3 member 1.

**Table 6:** Matches of peptides shared between *Aedes aegypti* and human proteins that bind with high affinity to MHC-II alleles associated with autoimmune diseases.

*Aedes aegypti* Protein	Matches	*Aedes aegypti* Peptides	Human Proteins	Alleles	≥80% Identity
Angiopoietin	113	23	Angiopoietin-related protein 2; angiopoietin-related protein 4; angiopoietin-related protein 6	HLA-DRB1*08:02	79
				HLA-DRB1*13:03	
				HLA-DRB1*14:02	
Ficolin	67	14	Angiopoietin-4; fibrinogen β chain; angiopoietin-related protein 6	HLA-DQA1*02:01/DQB1*02:01; HLA-DQA1*02:01/DQB1*02:02; HLA-DQA1*02:01/DQB1*02:04; HLA-DQA1*02:01/DQB1*02:05; HLA-DQA1*02:01/DQB1*02:06	36
				HLA-DQA1*04:01/DQB1*02:01; HLA-DQA1*04:01/DQB1*02:02; HLA-DQA1*04:01/DQB1*02:04; HLA-DQA1*04:01/DQB1*02:05; HLA-DQA1*04:01/DQB1*02:06; HLA-DQA1*04:01/DQB1*03:02	
				HLA-DQA1*04:02/DQB1*02:01; HLA-DQA1*04:02/DQB1*02:02; HLA-DQA1*04:02/DQB1*02:04; HLA-DQA1*04:02/DQB1*02:05; HLA-DQA1*04:02/DQB1*02:06; HLA-DQA1*04:02/DQB1*03:02	
				HLA-DQA1*05:01/DQB1*02:01; HLA-DQA1*05:01/DQB1*02:02; HLA-DQA1*05:01/DQB1*02:04; HLA-DQA1*05:01/DQB1*02:05; HLA-DQA1*05:01/DQB1*02:06	
				HLA-DRB1*04:05	
				HLA-DRB1*04:08	
				HLA-DRB1*04:10	
LTRIN	33	6	Forkhead box protein D3	HLA-DQA1*01:02/DQB1*03:01	0
				HLA-DQA1*02:01/DQB1*03:01	
				HLA-DQA1*04:01/DQB1*03:01; HLA-DQA1*04:01/DQB1*03:03	
				HLA-DQA1*04:02/DQB1*03:01; HLA-DQA1*04:02/DQB1*03:03; HLA-DQA1*04:02/DQB1*03:04	
				HLA-DQA1*05:01/DQB1*03:01; HLA-DQA1*05:01/DQB1*03:03; HLA-DQA1*05:01/DQB1*03:04	
Aldehyde dehydrogenase	25	16	Aldehyde dehydrogenase 3	HLA-DRB1*04:01	14
				HLA-DRB1*04:02	
				HLA-DRB1*04:04	
				HLA-DRB1*04:08	
				HLA-DRB1*04:10	
				HLA-DRB1*10:01	
Amylase	12	7	α-amylase 1A	HLA-DRB1*03:01	12
				HLA-DRB1*03:04	
				HLA-DRB1*03:08	
				HLA-DRB1*04:02	
				HLA-DRB1*14:02	
Venom allergen 5	9	3	Versican	HLA-DRB1*03:01	3
				HLA-DRB1*03:04	
				HLA-DRB1*03:07	
				HLA-DRB1*03:08	
6	259	69	10	46	144

LTRIN: lymphotoxin β receptor inhibitor.

**Table 7:** Frequency of matches for MHC-II alleles that bind peptides shared between *Aedes aegypti* and human proteins with high affinity.

Matches	MHC-II Alleles	Total Alleles
58	HLA-DRB1*13:03	1
37	HLA-DRB1*08:02	1
21	HLA-DRB1*14:02	1
13	HLA-DRB1*04:05	1
10	HLA-DRB1*10:01	1
7	HLA-DRB1*04:02	1
6	HLA-DQA1*04:01/DQB1*03:01; HLA-DQA1*04:02/DQB1*03:01; HLA-DRB1*04:04	3
5	HLA-DRB1*03:04; HLA-DRB1*03:08	2
4	HLA-DQA1*02:01/DQB1*02:05; HLA-DQA1*04:01/DQB1*02:05; HLA-DQA1*04:02/DQB1*02:05; HLA-DQA1*04:02/DQB1*03:04; HLA-DQA1*05:01/DQB1*02:05; HLA-DQA1*05:01/DQB1*03:01; HLA-DRB1*04:08; HLA-DRB1*04:10; HLA-DRB1*03:01	9
3	HLA-DQA1*01:02/DQB1*03:01; HLA-DQA1*02:01/DQB1*02:01; HLA-DQA1*02:01/DQB1*02:02; HLA-DQA1*02:01/DQB1*02:04; HLA-DQA1*02:01/DQB1*02:06; HLA-DQA1*05:01/DQB1*02:01; HLA-DQA1*05:01/DQB1*02:02; HLA-DQA1*05:01/DQB1*02:04; HLA-DQA1*05:01/DQB1*02:06	9
2	HLA-DQA1*02:01/DQB1*03:01; HLA-DQA1*04:01/DQB1*03:03; HLA-DQA1*04:02/DQB1*03:03; HLA-DQA1*05:01/DQB1*03:03; HLA-DQA1*05:01/DQB1*03:04	5
1	HLA-DQA1*04:01/DQB1*02:01; HLA-DQA1*04:01/DQB1*02:02; HLA-DQA1*04:01/DQB1*02:04; HLA-DQA1*04:01/DQB1*02:06; HLA-DQA1*04:01/DQB1*03:02; HLA-DQA1*04:02/DQB1*02:01; HLA-DQA1*04:02/DQB1*02:02; HLA-DQA1*04:02/DQB1*02:04; HLA-DQA1*04:02/DQB1*02:06; HLA-DQA1*04:02/DQB1*03:02; HLA-DRB1*04:01; HLA-DRB1*03:07	12

**Table 8:** Matches of peptides shared between *Aedes aegypti* and human proteins that bind with high affinity to MHC-I alleles associated with autoimmune diseases.

*Aedes aegypti* Protein	Matches	*Aedes aegypti* Peptides	Human Proteins	Alleles	Identical
Aldehyde dehydrogenase	10	3	Aldehyde dehydrogenase 3; INO80 complex subunit E	HLA-A*02:07; HLA-A*24:02; HLA-A*24:03; HLA-B*07:02; HLA-B*35:02; HLA-B*46:01; HLA-C*03:02; HLA-C*07:04; HLA-C*16:01	7
Ficolin	6	4	Angiopoietin-related protein 3; angiopoietin-related protein 6; ficolin	HLA-B*37:01; HLA-B*44:03; HLA-C*03:03; HLA-C*08:02; HLA-C*15:02; HLA-C*16:01	0
Angiopoietin	6	3	Angiopoietin-related protein 2; fibrinogen γ chain	HLA-A*24:03; HLA-C*06:02; HLA-C*07:01; HLA-C*07:02; HLA-C*07:04; HLA-C*08:02	2
Adenosine deaminase	6	2	Adenosine deaminase 2	HLA-C*03:03; HLA-C*06:02; HLA-C*15:02; HLA-C*16:01	6
α-glucosidase	6	1	SLC3A1	HLA-A*24:02; HLA-A*24:03; HLA-C*06:02; HLA-C*07:01; HLA-C*07:02; HLA-C*07:04	6
D7L1	3	2	Pro eosinophil major basic protein	HLA-B*39:06; HLA-C*01:02; HLA-C*06:02	0
NeSt1	2	1	SH3 domain-binding protein 5-like	HLA-A*02:07; HLA-C*15:02	0
AgBR1	1	1	Chitinase-3-like protein 1	HLA-A*24:02	0
Lysozyme	1	1	L-dopachrome tautomerase	HLA-A*02:07	0
Mucin 6	1	1	PI-binding clathrin assembly protein	HLA-C*01:02	1
10	43	19	14	19	22

AgBR: *Aedes aegypti* bacteria-responsive protein; NeSt: neutrophil-stimulating factor; PI: phosphatidylinositol; SLC3A1: solute carrier family 3 member 1.

**Table 9:** Frequency of matches for MHC-I alleles that bind peptides shared between *Aedes aegypti* and human proteins with high affinity.

Matches	MHC-II Alleles	Total Alleles
4	HLA-A*02:07; HLA-C*06:02; HLA-C*15:02; HLA-C*16:01	4
3	HLA-A*24:02; HLA-A*24:03; HLA-C*07:04	3
2	HLA-C*01:02; HLA-C*03:03; HLA-C*07:01; HLA-C*07:02; HLA-C*08:02	5
1	HLA-B*07:02; HLA-B*35:02; HLA-B*37:01; HLA-B*39:06; HLA-B*44:03; HLA-B*46:01; HLA-C*03:02	7

**Table 10:** IEDB peptides that contain sequences shared between *Aedes aegypti* salivary proteins and human proteins.

Human Protein	IEDB Peptide	Peptide ID	Validation	Reference
Fibrinogen β chain	GGWTVIQNRQDG	961381	B cell activation; association to RA	[121]
	GWTVIQNRQDGS	962133		
	WTVIQNRQDGSV	968636		
Aldehyde dehydrogenase 3	EIFGPILPIVNV	2244697	Presentation bound to MHC-I	[122,123,124]
	QDEIFGPILP	1266040		
	LPFGGVGASGMGRY	621205		
Fibrinogen γ chain	LDGSVDFK	1028523	Processing from tumor antigen; presentation bound to MHC-I alleles HLA-C*16:01 and HLA-A*34:02	[125,126,127]
	RLDGSVDFK	991140		
Angiopoietin-related protein 6	YHGDAGDSL	450124	Presentation bound to MHC-I alleles HLA-B*15:10, HLA-B*38:01 and HLA-B*38:02; association to T1D	[125,128,129,130,131,132]
α-amylase 1A	ALVFVDNHDNQ	2185722	Presentation bound to MHC-I	[133]
Adenosine deaminase 2	NQVLKLVSDLRNHP	2038620	Presentation bound to MHC-II	[134]
L-dopachrome tautomerase	ALVGLFVLL	2961	Processing from tumor antigen; presentation bound to MHC-I allele HLA-A*02:01	[135]
Pro eosinophil major basic protein	KLPLLLAL	1393024	Presentation bound to MHC-II allele HLA-DRB1*15:01; association to MS	[136]
TNRC6A	QQQQQQQQPQQQQPQ	177936	B cell activation; association to SLE	[137]

TNRC6A: trinucleotide repeat containing adaptor 6A; RA: rheumatoid arthritis; T1D: type 1 diabetes; SLE: systemic lupus erythematosus.

**Table 11:** Localization and function of human proteins that share peptides with *Aedes aegypti* salivary proteins.

Localization	Protein	Tissue/Cell Type Distribution	Function	Reference
Nucleus	Ataxin 1	Neurons, oligodendrocytes	Transcriptional regulation	[142]
	CHD-1	Ubiquitous	Chromatin remodeling	[143]
	Forkhead box protein D3	Schwann cells, melanocytes	Transcriptional regulation	[144]
	Histone-lysine N-methyltransferase 2D	Ubiquitous	Chromatin remodeling	[145]
	INO80 complex subunit E	Ubiquitous	Chromatin remodeling	[146]
	Mediator complex subunit 12	Ubiquitous	Transcriptional regulation	[147]
	MIDEAS	Ubiquitous	Histone deacetylation	[148]
	N-lysine methyltransferase SETD6	Ubiquitous	Transcriptional regulation	[149]
	Nuclear receptor corepressor 2	Ubiquitous	Transcriptional repression	[150]
	Nucleophosmin	Ubiquitous	Ribosome nuclear export	[151]
	Transcriptional activator MN1	Ubiquitous	Transcriptional activation	[152]
	Tumor protein 63	Epithelial cells	Transcriptional regulation	[153]
	Zinc finger homeobox protein 3	Ubiquitous	Transcriptional regulation	[154]
	Zinc finger MYM-type protein 4	Ubiquitous	Transcriptional regulation	[155]
	Zinc finger RNA binding protein	Ubiquitous	Regulation of RNA metabolism	[156]
Cytoplasm	Ataxin 2	CNS	Regulation of EGFR endocytosis	[157]
	Tyrosine-protein kinase	Ubiquitous	Regulation of cytoskeleton function	[158]
	GRB10-interacting GYF protein 2	Ubiquitous	Translational repression	[159]
	Huntingtin	Brain	Scaffolding in vesicle transport	[160]
	L-dopachrome tautomerase	Melanocytes	Enzyme in melanin synthesis	[161]
	PAT1 homolog 2	Oocytes	Translational repression	[162]
	Rho GEF 37	Ubiquitous	Signal transduction	[163]
	SH3 domain-binding protein 5-like	Ubiquitous	Signal transduction	[164]
	TNRC6A	Ubiquitous	Translational repression	[165]
	Tubulin α-8		Constituent of microtubules	
ER	Aldehyde dehydrogenase 3	Liver	Enzyme in lipid metabolism	[166]
	ER to nucleus signaling 1	Ubiquitous	Unfolded protein response	[167]
	SCAPER	Ubiquitous	Cell cycle regulation	[168]
Mitochondria	DNA polymerase subunit γ-1	Ubiquitous	Replication of mitochondrial DNA	[169]
	NADH dehydrogenase subunit 2	Brain, heart muscle	Enzyme in respiratory chain	[170]
Cell membrane	AP2-associated protein kinase 1	Neurons, T cells, NK cells	Regulation of endocytosis	[171]
	DC-STAMP domain-containing protein 2	Sperm cells	Sperm–egg fusion	[172]
	Dopamine receptor D4	Retina, neurons	Neuronal signaling	[173]
	PI-binding clathrin assembly protein	Ubiquitous	Adapter protein in endocytosis	[174]
	RPTP-α	Ubiquitous	Focal adhesion formation	[175]
	SLC3A1	Kidney and small intestine	Amino acid transporter	[176]
	Tensin-1	Ubiquitous	Fibrillar adhesion formation	[177]
Secreted	Adenosine deaminase 2	Blood	Regulation of adenosine function	[178]
	α-amylase 1A	Saliva	Carbohydrate digestion	[179]
	Angiopoietin-4	Blood	Regulation of angiogenesis	[180]
	Angiopoietin-related protein 2	Blood	Promotes inflammation	[181]
	Angiopoietin-related protein 3	Blood	Regulation of lipid and glucose metabolism	[182]
	Angiopoietin-related protein 4	Blood, ECM	Regulation of lipid metabolism and endothelial cell function	[183]
	Angiopoietin-related protein 6	Blood	Wound healing	[184]
	Cartilage oligomeric matrix protein	ECM	Structural integrity of cartilage	[185]
	Chitinase-3-like protein 1	Blood	Promotes inflammation	[186]
	Fibrinogen β chain	Blood	Clot formation	[187]
	Fibrinogen γ chain	Blood	Clot formation	[188]
	Fibrinogen-like 1	Blood	Inhibition of T cell activation	[189]
	Ficolin	Blood	Pattern recognition receptor	[190]
	Pro eosinophil major basic protein	Blood	Cytotoxin and helminthotoxin	[191]
	Versican	ECM	Regulation of cell migration	[192]

CHD-1: chromodomain-helicase-DNA-binding protein-1; MIDEAS: mitotic deacetylase-associated SANT domain protein; EGFR: epidermal growth factor receptor; GEF: guanine nucleotide exchange factor; TNRC6A: trinucleotide repeat containing adaptor 6A; SCAPER: S phase cyclin A-associated protein in the endoplasmic reticulum; ER: endoplasmic reticulum; PI: phosphatidylinositol; CNS: central nervous system; RPTP: receptor-type tyrosine-protein phosphatase; SLC3A1: solute carrier family 3 member 1.

## Data Availability

The original contributions presented in this study are included in the article/Supplementary Materials. Further inquiries can be directed to the corresponding author.

## Supplementary Materials

The following supporting information can be downloaded at: https://www.mdpi.com/article/10.3390/proteomes13040056/s1, Supplementary File S1. Identical peptide sequences shared between *Aedes aegypti* salivary proteins and human proteins. Supplementary File S2. Matching peptides in *Aedes aegypti* salivary proteins and human proteins binding with high affinity to MHC-II alleles associated to autoimmunity. Supplementary File S3. Matching peptides in *Aedes aegypti* salivary proteins and human proteins binding with high affinity to MHC-I alleles associated to autoimmunity.

## Abbreviations

The following abbreviations are used in this manuscript:

AaVA-1	*Aedes aegypti* venom allergen-1
AeMOPE	*Aedes*-specific modulatory peptide
AgBR	*Aedes aegypti* bacteria-responsive protein
AIH	Autoimmune hepatitis
CD	Crohn’s disease
CHD-1	Chromodomain-helicase-DNA-binding protein-1
CHIKV	Chikungunya virus
CLIP	Cytoplasmic linker protein
CNS	Central nervous system
DENV	Dengue virus
ECM	Extracellular matrix
EGFR	Epidermal growth factor receptor
ER	Endoplasmic reticulum
GD	Graves’ disease
GEF	Guanine nucleotide exchange factor
ITP	Immune thrombocytopenic purpura
LTRIN	Lymphotoxin β receptor inhibitor
MG	Myasthenia gravis
MIDEAS	Mitotic deacetylase-associated SANT domain protein
MS	Multiple sclerosis
NeSt	Neutrophil-stimulating factor
PAMP	Pathogen-associated molecular pattern
PE	Phosphatidylethanolamine
PI	Phosphatidylinositol
PR	Polymyalgia rheumatica
RA	Rheumatoid arthritis
RPTP	Receptor-type tyrosine-protein phosphatase
SCAPER	S phase cyclin A-associated protein in the endoplasmic reticulum
SLC3A1	Solute carrier family 3 member 1
SLE	Systemic lupus erythematosus
SS	Sjögren’s syndrome
T1D	Type 1 diabetes
TNRC6A	Trinucleotide repeat containing adaptor 6A
UC	Ulcerative colitis
ZIKV	Zika virus

## References

[1] Souza-Neto J.A., Powell J.R., Bonizzoni M. (2019). Aedes aegypti vector competence studies: A review. Infect. Genet. Evol..

[2] Alonso-Palomares L.A., Moreno-García M., Lanz-Mendoza H., Salazar M.I. (2018). Molecular Basis for Arbovirus Transmission by Aedes aegypti Mosquitoes. Intervirology.

[3] Khan Z.A., Yadav M.K., Lim D.-W., Kim H., Wang J.-H., Ansari A. (2024). Viral-host molecular interactions and metabolic modulation: Strategies to inhibit flaviviruses pathogenesis. World J. Virol..

[4] Aguilar-Briseño J.A., Moser J., Rodenhuis-Zybert I.A. (2020). Understanding immunopathology of severe dengue: Lessons learnt from sepsis. Curr. Opin. Virol..

[5] Christian K.M., Song H., Ming G.-L. (2019). Pathophysiology and Mechanisms of Zika Virus Infection in the Nervous System. Annu. Rev. Neurosci..

[6] Cherie T.J.J., Choong C.S.H., Abid M.B., Weber M.W., Yap E.S., Seneviratne S.L., Abeysuriya V., de Mel S. (2024). Immuno-Haematologic Aspects of Dengue Infection: Biologic Insights and Clinical Implications. Viruses.

[7] Lardo S., Soesatyo M.H., Juffrie J., Umniyati S.R. (2018). The Autoimmune Mechanism in Dengue Hemorrhagic Fever. Acta Med. Indones..

[8] Martina B.E.E., Koraka P., Osterhaus A.D.M.E. (2009). Dengue virus pathogenesis: An integrated view. Clin. Microbiol. Rev..

[9] Srikiatkhachorn A., Mathew A., Rothman A.L. (2017). Immune-mediated cytokine storm and its role in severe dengue. Semin. Immunopathol..

[10] Lin C.-F., Lei H.-Y., Shiau A.-L., Liu C.-C., Liu H.-S., Yeh T.-M., Chen S.-H., Lin Y.-S. (2003). Antibodies from dengue patient sera cross-react with endothelial cells and induce damage. J. Med. Virol..

[11] Lin C.-F., Lei H.-Y., Shiau A.-L., Liu H.-S., Yeh T.-M., Chen S.-H., Liu C.-C., Chiu S.-C., Lin Y.-S. (2002). Endothelial cell apoptosis induced by antibodies against dengue virus nonstructural protein 1 via production of nitric oxide. J. Immunol..

[12] Chungue E., Poli L., Roche C., Gestas P., Glaziou P., Markoff L.J. (1994). Correlation between detection of plasminogen cross-reactive antibodies and hemorrhage in dengue virus infection. J. Infect. Dis..

[13] Wan S.-W., Lin C.-F., Yeh T.-M., Liu C.-C., Liu H.-S., Wang S., Ling P., Anderson R., Lei H.-Y., Lin Y.-S. (2013). Autoimmunity in dengue pathogenesis. J. Formos. Med. Assoc..

[14] Falconar A.K. (1997). The dengue virus nonstructural-1 protein (NS1) generates antibodies to common epitopes on human blood clotting, integrin/adhesin proteins and binds to human endothelial cells: Potential implications in haemorrhagic fever pathogenesis. Arch. Virol..

[15] Cheng H.-J., Luo Y.-H., Wan S.-W., Lin C.-F., Wang S.-T., Hung N.T., Liu C.-C., Ho T.-S., Liu H.-S., Yeh T.-M. (2015). Correlation between serum levels of anti-endothelial cell autoantigen and anti-dengue virus nonstructural protein 1 antibodies in dengue patients. Am. J. Trop. Med. Hyg..

[16] Ghorai T., Sarkar A., Roy A., Bhowmick B., Nayak D., Das S. (2024). Role of auto-antibodies in the mechanisms of dengue pathogenesis and its progression: A comprehensive review. Arch. Microbiol..

[17] Rajadhyaksha A., Mehra S. (2012). Dengue fever evolving into systemic lupus erythematosus and lupus nephritis: A case report. Lupus.

[18] Jardim D.L.F., Tsukumo D.M.L., Angerami R.N., Carvalho Filho M.A., de Saad M.J.A. (2012). Autoimmune features caused by dengue fever: A case report. Braz. J. Infect. Dis..

[19] Harris V.K., Danda D., Murali N.S., Das P.K., Abraham M., Cherian A.M., Chandy M. (2000). Unusual association of Kikuchi’s disease and dengue virus infection evolving into systemic lupus erythematosus. J. Indian Med. Assoc..

[20] Puccioni-Sohler M., Ornelas A.M.M., de Souza A.S., Cabral-Castro M.J., Ramos J.T.M.A., Rosadas C., Salgado M.C.F., Castiglione A.A., Ferry F., Peralta J.M. (2017). First report of persistent dengue-1-associated autoimmune neurological disturbance: Neuromyelitis optica spectrum disorder. J. Neurovirol..

[21] Rivera-Correa J., de Siqueira I.C., Mota S., do Rosário M.S., Pereira de Jesus P.A., Alcantara L.C.J., Ernst J.D., Rodriguez A. (2019). Anti-ganglioside antibodies in patients with Zika virus infection-associated Guillain-Barré Syndrome in Brazil. PLoS Negl. Trop. Dis..

[22] Acosta-Ampudia Y., Monsalve D.M., Castillo-Medina L.F., Rodríguez Y., Pacheco Y., Halstead S., Willison H.J., Anaya J.-M., Ramírez-Santana C. (2018). Autoimmune Neurological Conditions Associated with Zika Virus Infection. Front. Mol. Neurosci..

[23] Anaya J.-M., Rodríguez Y., Monsalve D.M., Vega D., Ojeda E., González-Bravo D., Rodríguez-Jiménez M., Pinto-Díaz C.A., Chaparro P., Gunturiz M.L. (2017). A comprehensive analysis and immunobiology of autoimmune neurological syndromes during the Zika virus outbreak in Cúcuta, Colombia. J. Autoimmun..

[24] Payus A.O., Ibrahim A., Lin C.L.S., Jan T.H. (2022). Sensory Predominant Guillain-Barré Syndrome Concomitant with Dengue Infection: A Case Report. Case Rep. Neurol..

[25] Dalugama C., Shelton J., Ekanayake M., Gawarammana I.B. (2018). Dengue fever complicated with Guillain-Barré syndrome: A case report and review of the literature. J. Med. Case Rep..

[26] Sreelakshmi V., Pattanaik A., Marate S., Mani R.S., Pai A.R., Mukhopadhyay C. (2024). Guillain-barré syndrome (GBS) with antecedent chikungunya infection: A case report and literature review. Neurol. Res. Pract..

[27] Oehler E., Watrin L., Larre P., Leparc-Goffart I., Lastere S., Valour F., Baudouin L., Mallet H., Musso D., Ghawche F. (2014). Zika virus infection complicated by Guillain-Barre syndrome—Case report, French Polynesia, December 2013. Euro. Surveill..

[28] Rivera-Concepción J.R., Betancourt J.P., Cerra J., Reyes E. (2018). The Zika Virus: An Association to Guillain-Barré Syndrome in the United States—A Case Report. P. R. Health Sci. J..

[29] Mancera-Páez O., Román G.C., Pardo-Turriago R., Rodríguez Y., Anaya J.-M. (2018). Concurrent Guillain-Barré syndrome, transverse myelitis and encephalitis post-Zika: A case report and review of the pathogenic role of multiple arboviral immunity. J. Neurol. Sci..

[30] Bautista L.E. (2019). Zika virus infection and risk of Guillain-Barré syndrome: A meta-analysis. J. Neurol. Sci..

[31] Simmer P.E., Powell V., Hoch V., Noblett C., Eckert P., Abdelmaseeh P. (2025). Ascending Trouble: Guillain-Barré-Like Syndrome Due to West Nile Virus. Cureus.

[32] Beshai R., Bibawy D., Bibawy J. (2020). Guillain-Barré Syndrome Secondary to West Nile Virus in New York City. Case Rep. Infect. Dis..

[33] Karagianni P., Alexopoulos H., Sourdi A., Papadimitriou D., Dimitrakopoulos A.N., Moutsopoulos H.M. (2019). West Nile Virus infection triggering autoimmune encephalitis: Pathophysiological and therapeutic implications. Clin. Immunol..

[34] Theofilopoulos A.N., Kono D.H., Baccala R. (2017). The multiple pathways to autoimmunity. Nat. Immunol..

[35] Marson A., Housley W.J., Hafler D.A. (2015). Genetic basis of autoimmunity. J. Clin. Investig..

[36] Rosenblum M.D., Remedios K.A., Abbas A.K. (2015). Mechanisms of human autoimmunity. J. Clin. Investig..

[37] Cusick M.F., Libbey J.E., Fujinami R.S. (2012). Molecular mimicry as a mechanism of autoimmune disease. Clin. Rev. Allergy Immunol..

[38] Rojas M., Restrepo-Jiménez P., Monsalve D.M., Pacheco Y., Acosta-Ampudia Y., Ramírez-Santana C., Leung P.S.C., Ansari A.A., Gershwin M.E., Anaya J.-M. (2018). Molecular mimicry and autoimmunity. J. Autoimmun..

[39] Suliman B.A. (2024). Potential clinical implications of molecular mimicry-induced autoimmunity. Immun. Inflamm. Dis..

[40] Kalil J., Guilherme L. (2020). Rheumatic Fever: A Model of Autoimmune Disease due to Molecular Mimicry between Human and Pathogen Proteins. Crit. Rev. Immunol..

[41] Shahrizaila N., Yuki N. (2011). Guillain-barré syndrome animal model: The first proof of molecular mimicry in human autoimmune disorder. J. Biomed Biotechnol..

[42] Poole B.D., Scofield R.H., Harley J.B., James J.A. (2006). Epstein-Barr virus and molecular mimicry in systemic lupus erythematosus. Autoimmunity.

[43] Goh L., Kerkar N. (2024). Hepatitis C Virus and Molecular Mimicry. Pathogens.

[44] Arévalo-Cortés A., Rodriguez-Pinto D., Aguilar-Ayala L. (2024). Evidence for Molecular Mimicry between SARS-CoV-2 and Human Antigens: Implications for Autoimmunity in COVID-19. Autoimmune Dis..

[45] Hussein H.M., Rahal E.A. (2019). The role of viral infections in the development of autoimmune diseases. Crit. Rev. Microbiol..

[46] Cunningham M.W. (2019). Molecular Mimicry, Autoimmunity, and Infection: The Cross-Reactive Antigens of Group A Streptococci and their Sequelae. Microbiol. Spectr..

[47] Szymula A., Rosenthal J., Szczerba B.M., Bagavant H., Fu S.M., Deshmukh U.S. (2014). T cell epitope mimicry between Sjögren’s syndrome Antigen A (SSA)/Ro60 and oral, gut, skin and vaginal bacteria. Clin. Immunol..

[48] Zhang W., Reichlin M. (2008). A possible link between infection with burkholderia bacteria and systemic lupus erythematosus based on epitope mimicry. Clin. Dev. Immunol..

[49] Muthye V., Wasmuth J.D. (2023). Proteome-wide comparison of tertiary protein structures reveals molecular mimicry in Plasmodium-human interactions. Front. Parasitol..

[50] Emiliani Y., Muzi G., Sánchez A., Sánchez J., Munera M. (2022). Prediction of molecular mimicry between proteins from Trypanosoma sp. and human antigens associated with systemic lupus erythematosus. Microb. Pathog..

[51] Trier N.H., Houen G. (2023). Antibody Cross-Reactivity in Auto-Immune Diseases. Int. J. Mol. Sci..

[52] Kammer A.R., van der Burg S.H., Grabscheid B., Hunziker I.P., Kwappenberg K.M., Reichen J., Melief C.J., Cerny A. (1999). Molecular mimicry of human cytochrome P450 by hepatitis C virus at the level of cytotoxic T cell recognition. J. Exp. Med..

[53] Johnston A., Gudjonsson J.E., Sigmundsdottir H., Love T.J., Valdimarsson H. (2004). Peripheral blood T cell responses to keratin peptides that share sequences with streptococcal M proteins are largely restricted to skin-homing CD8^+^ T cells. Clin. Exp. Immunol..

[54] Liu I.-J., Chiu C.-Y., Chen Y.-C., Wu H.-C. (2011). Molecular mimicry of human endothelial cell antigen by autoantibodies to nonstructural protein 1 of dengue virus. J. Biol. Chem..

[55] Cheng H.-J., Lin C.-F., Lei H.-Y., Liu H.-S., Yeh T.-M., Luo Y.-H., Lin Y.-S. (2009). Proteomic analysis of endothelial cell autoantigens recognized by anti-dengue virus nonstructural protein 1 antibodies. Exp. Biol. Med..

[56] Cheng H.-J., Lei H.-Y., Lin C.-F., Luo Y.-H., Wan S.-W., Liu H.-S., Yeh T.-M., Lin Y.-S. (2009). Anti-dengue virus nonstructural protein 1 antibodies recognize protein disulfide isomerase on platelets and inhibit platelet aggregation. Mol. Immunol..

[57] Chang H.-H., Shyu H.-F., Wang Y.-M., Sun D.-S., Shyu R.-H., Tang S.-S., Huang Y.-S. (2002). Facilitation of cell adhesion by immobilized dengue viral nonstructural protein 1 (NS1): Arginine-glycine-aspartic acid structural mimicry within the dengue viral NS1 antigen. J. Infect. Dis..

[58] Huang Y.H., Chang B.I., Lei H.Y., Liu H.S., Liu C.C., Wu H.L., Yeh T.M. (1997). Antibodies against dengue virus E protein peptide bind to human plasminogen and inhibit plasmin activity. Clin. Exp. Immunol..

[59] Lin Y.-S., Yeh T.-M., Lin C.-F., Wan S.-W., Chuang Y.-C., Hsu T.-K., Liu H.-S., Liu C.-C., Anderson R., Lei H.-Y. (2011). Molecular mimicry between virus and host and its implications for dengue disease pathogenesis. Exp. Biol. Med..

[60] Lucchese G., Kanduc D. (2016). Zika virus and autoimmunity: From microcephaly to Guillain-Barré syndrome, and beyond. Autoimmun. Rev..

[61] França L.C., Fontes-Dantas F.L., Garcia D.G., de Araújo A.D., da Costa Gonçalves J.P., da Silav Rêgo C.C., da Silva E.V., do Nascimento O.J.M., Lopes F.C.R., Herlinger A.L. (2023). Molecular mimicry between Zika virus and central nervous system inflammatory demyelinating disorders: The role of NS5 Zika virus epitope and PLP autoantigens. Arq. Neuropsiquiatr..

[62] Nasirudeen A.M.A., Wong H.H., Thien P., Xu S., Lam K.-P., Liu D.X. (2011). RIG-I, MDA5 and TLR3 synergistically play an important role in restriction of dengue virus infection. PLoS Negl. Trop. Dis..

[63] Ye S., Liang Y., Chang Y., Lai B., Zhong J. (2025). Dengue Virus Replicative-Form dsRNA Is Recognized by Both RIG-I and MDA5 to Activate Innate Immunity. J. Med. Virol..

[64] Suthar M.S., Aguirre S., Fernandez-Sesma A. (2013). Innate Immune Sensing of Flaviviruses. PLoS Pathog..

[65] Pingen M., Schmid M.A., Harris E., McKimmie C.S. (2017). Mosquito Biting Modulates Skin Response to Virus Infection. Trends Parasitol..

[66] Demeure C.E., Brahimi K., Hacini F., Marchand F., Péronet R., Huerre M., St.-Mezard P., Nicolas J.-F., Brey P., Delespesse G. (2005). Anopheles Mosquito Bites Activate Cutaneous Mast Cells Leading to a Local Inflammatory Response and Lymph Node Hyperplasia1. J. Immunol..

[67] Pingen M., Bryden S.R., Pondeville E., Schnettler E., Kohl A., Merits A., Fazakerley J.K., Graham G.J., McKimmie C.S. (2016). Host Inflammatory Response to Mosquito Bites Enhances the Severity of Arbovirus Infection. Immunity.

[68] Guerrero D., Cantaert T., Missé D. (2020). Aedes Mosquito Salivary Components and Their Effect on the Immune Response to Arboviruses. Front. Cell. Infect. Microbiol..

[69] Ader D.B., Celluzzi C., Bisbing J., Gilmore L., Gunther V., Peachman K.K., Rao M., Barvir D., Sun W., Palmer D.R. (2004). Modulation of Dengue Virus Infection of Dendritic Cells by Aedes aegypti Saliva. Viral Immunol..

[70] Schneider B.S., Soong L., Zeidner N.S., Higgs S. (2004). Aedes aegypti Salivary Gland Extracts Modulate Anti-Viral and TH1/TH2 Cytokine Responses to Sindbis Virus Infection. Viral Immunol..

[71] Schmid M.A., Glasner D.R., Shah S., Michlmayr D., Kramer L.D., Harris E. (2016). Mosquito Saliva Increases Endothelial Permeability in the Skin, Immune Cell Migration, and Dengue Pathogenesis during Antibody-Dependent Enhancement. PLoS Pathog..

[72] Martin-Martin I., Kern O., Brooks S., Smith L.B., Valenzuela-Leon P.C., Bonilla B., Ackerman H., Calvo E. (2021). Biochemical characterization of AeD7L2 and its physiological relevance in blood feeding in the dengue mosquito vector, Aedes aegypti. FEBS J..

[73] Martin-Martin I., Valenzuela Leon P.C., Amo L., Shrivastava G., Iniguez E., Aryan A., Brooks S., Kojin B.B., Williams A.E., Bolland S. (2022). Aedes aegypti sialokinin facilitates mosquito blood feeding and modulates host immunity and vascular biology. Cell Rep..

[74] Ribeiro J.M. (1992). Characterization of a vasodilator from the salivary glands of the yellow fever mosquito Aedes aegypti. J. Exp. Biol..

[75] Peng Z., Rasic N., Liu Y., Simons F.E.R. (2002). Mosquito saliva–specific IgE and IgG antibodies in 1059 blood donors. J. Allergy Clin. Immunol..

[76] Londoño-Rentería B., Cárdenas J.C., Giovanni J.E., Cárdenas L., Villamizar P., Rolón J., Chisenhall D.M., Christofferson R.C., Carvajal D.J., Pérez O.G. (2015). Aedes aegypti anti-salivary gland antibody concentration and dengue virus exposure history in healthy individuals living in an endemic area in Colombia. Biomedica.

[77] Chea S., Willen L., Nhek S., Ly P., Tang K., Oristian J., Salas-Carrillo R., Ponce A., Leon P.C.V., Kong D. (2024). Antibodies to Aedes aegypti D7L salivary proteins as a new serological tool to estimate human exposure to Aedes mosquitoes. Front. Immunol..

[78] Mathieu-Daudé F., Claverie A., Plichart C., Boulanger D., Mphande F.A., Bossin H.C. (2018). Specific human antibody responses to Aedes aegypti and Aedes polynesiensis saliva: A new epidemiological tool to assess human exposure to disease vectors in the Pacific. PLoS Negl. Trop. Dis..

[79] Li N., Aoki V., Liu Z., Prisayanh P., Valenzuela J.G., Diaz L.A. (2022). From Insect Bites to a Skin Autoimmune Disease: A Conceivable Pathway to Endemic Pemphigus Foliaceus. Front. Immunol..

[80] Ribeiro J.M.C., Martin-Martin I., Arcà B., Calvo E. (2016). A Deep Insight into the Sialome of Male and Female Aedes aegypti Mosquitoes. PLoS ONE.

[81] Ribeiro J.M.C., Arcà B., Lombardo F., Calvo E., Phan V.M., Chandra P.K., Wikel S.K. (2007). An annotated catalogue of salivary gland transcripts in the adult female mosquito, Aedes aegypti. BMC Genom..

[82] Wasinpiyamongkol L., Patramool S., Luplertlop N., Surasombatpattana P., Doucoure S., Mouchet F., Séveno M., Remoue F., Demettre E., Brizard J.-P. (2010). Blood-feeding and immunogenic Aedes aegypti saliva proteins. Proteomics.

[83] Gavor E., Choong Y.K., Liu Y., Pompon J., Ooi E.E., Mok Y.K., Liu H., Kini R.M., Sivaraman J. (2022). Identification of Aedes aegypti salivary gland proteins interacting with human immune receptor proteins. PLoS Negl. Trop. Dis..

[84] Schumacher T.N., De Bruijn M.L., Vernie L.N., Kast W.M., Melief C.J., Neefjes J.J., Ploegh H.L. (1991). Peptide selection by MHC class I molecules. Nature.

[85] Rammensee H.G., Friede T., Stevanoviíc S. (1995). MHC ligands and peptide motifs: First listing. Immunogenetics.

[86] Renaudineau Y., Charras A., Natoli V., Congy-Jolivet N., Haldenby S., Liu X., Fang Y., Smith E.M., Beresford M.W., Hedrich C.M. (2025). Across ancestries, HLA-B∗08:01∼DRB1∗03:01 (DR3) and HLA-DQA∗01:02 (DR2) increase the risk to develop juvenile-onset systemic lupus erythematosus through low complement C4 levels. J. Transl. Autoimmun..

[87] Butler-Laporte G., Farjoun J., Nakanishi T., Lu T., Abner E., Chen Y., Hultström M., Metspalu A., Milani L., Mägi R. (2023). HLA allele-calling using multi-ancestry whole-exome sequencing from the UK Biobank identifies 129 novel associations in 11 autoimmune diseases. Commun. Biol..

[88] Al Naqbi H., Mawart A., Alshamsi J., Al Safar H., Tay G.K. (2021). Major histocompatibility complex (MHC) associations with diseases in ethnic groups of the Arabian Peninsula. Immunogenetics.

[89] Fernando M.M.A., Stevens C.R., Walsh E.C., De Jager P.L., Goyette P., Plenge R.M., Vyse T.J., Rioux J.D. (2008). Defining the role of the MHC in autoimmunity: A review and pooled analysis. PLoS Genet..

[90] Erlich H., Valdes A.M., Noble J., Carlson J.A., Varney M., Concannon P., Mychaleckyj J.C., Todd J.A., Bonella P., Fear A.L. (2008). HLA DR-DQ haplotypes and genotypes and type 1 diabetes risk: Analysis of the type 1 diabetes genetics consortium families. Diabetes.

[91] Pirie F.J., Hammond M.G., Motala A.A., Omar M.A. (2001). HLA class II antigens in South African Blacks with type I diabetes. Tissue Antigens.

[92] Heward J.M., Allahabadia A., Daykin J., Carr-Smith J., Daly A., Armitage M., Dodson P.M., Sheppard M.C., Barnett A.H., Franklyn J.A. (1998). Linkage disequilibrium between the human leukocyte antigen class II region of the major histocompatibility complex and Graves’ disease: Replication using a population case control and family-based study. J. Clin. Endocrinol. Metab..

[93] Hernández-Doño S., Jakez-Ocampo J., Márquez-García J.E., Ruiz D., Acuña-Alonzo V., Lima G., Llorente L., Tovar-Méndez V.H., García-Silva R., Granados J. (2021). Heterogeneity of Genetic Admixture Determines SLE Susceptibility in Mexican. Front. Genet..

[94] Al-Harbi E.M., Abbassi A.-J., Tamim H., al-Jenaidi F., Kooheji M., Kamal M., al-Mahroos S., al-Nasir F., Motala A.A., Almawi W.Y. (2004). Specific HLA-DRB and -DQB alleles and haplotypes confer disease susceptibility or resistance in Bahraini type 1 diabetes patients. Clin. Diagn. Lab. Immunol..

[95] Varade J., Wang N., Lim C.K., Zhang T., Zhang Y., Liu X., Piehl F., Matell R., Cao H., Xu X. (2018). Novel genetic loci associated HLA-B*08:01 positive myasthenia gravis. J. Autoimmun..

[96] Le T.T.V., Vuong T.T.B., Ong T.P., Do M.D. (2022). Allele frequency and the associations of HLA-DRB1 and HLA-DQB1 polymorphisms with pemphigus subtypes and disease severity. Medicine.

[97] Feng X., Li W., Song J., Liu X., Gu Y., Yan C., Wu H., Xi J., Zhou S., Zhao C. (2019). HLA typing using next-generation sequencing for Chinese juvenile- and adult-onset myasthenia gravis patients. J. Clin. Neurosci..

[98] de Almeida D.E., Ling S., Holoshitz J. (2011). New insights into the functional role of the rheumatoid arthritis shared epitope. FEBS Lett..

[99] van Drongelen V., Holoshitz J. (2017). Human Leukocyte Antigen-Disease Associations in Rheumatoid Arthritis. Rheum. Dis. Clin. N. Am..

[100] Oliveira L.C., Porta G., Marin M.L.C., Bittencourt P.L., Kalil J., Goldberg A.C. (2011). Autoimmune hepatitis, HLA and extended haplotypes. Autoimmun. Rev..

[101] Jun K.R., Choi S.E., Cha C.H., Oh H.B., Heo Y.S., Ahn H.Y., Lee K.J. (2007). Meta-analysis of the association between HLA-DRB1 allele and rheumatoid arthritis susceptibility in Asian populations. J. Korean Med. Sci..

[102] Gough S.C.L., Simmonds M.J. (2007). The HLA Region and Autoimmune Disease: Associations and Mechanisms of Action. Curr. Genom..

[103] Patsopoulos N.A., Barcellos L.F., Hintzen R.Q., Schaefer C., van Duijn C.M., Noble J.A., Raj T., Gourraud P.-A., IMSGC, ANZgene (2013). Fine-mapping the genetic association of the major histocompatibility complex in multiple sclerosis: HLA and non-HLA effects. PLoS Genet..

[104] Hachicha H., Kammoun A., Mahfoudh N., Marzouk S., Feki S., Fakhfakh R., Fourati H., Haddouk S., Frikha F., Gaddour L. (2018). Human leukocyte antigens-DRB1*03 is associated with systemic lupus erythematosus and anti-SSB production in South Tunisia. Int. J. Health Sci..

[105] Jean S., Quelvennec E., Alizadeh M., Guggenbuhl P., Birebent B., Perdriger A., Grosbois B., Pawlotsky P.Y., Semana G. (1998). DRB1*15 and DRB1*03 extended haplotype interaction in primary Sjögren’s syndrome genetic susceptibility. Clin. Exp. Rheumatol..

[106] Yeo T.W., De Jager P.L., Gregory S.G., Barcellos L.F., Walton A., Goris A., Fenoglio C., Ban M., Taylor C.J., Goodman R.S. (2007). A second major histocompatibility complex susceptibility locus for multiple sclerosis. Ann. Neurol..

[107] Kikili C.İ., Kivanç D., Ortaboz D., Şentürk Çiftçi H., Özbalak M.M., Yenerel M.N., Nalçaci M., Ar M.C., Oğuz F.S., Beşişik S.K. (2024). Identification of HLA alleles involved in immune thrombotic thrombocytopenic purpura patients from Turkey. Blood Coagul. Fibrinolysis..

[108] Stasiak M., Zawadzka-Starczewska K., Tymoniuk B., Stasiak B., Lewiński A. (2023). Significance of HLA in the development of Graves’ orbitopathy. Genes Immun..

[109] Sirikong M., Tsuchiya N., Chandanayingyong D., Bejrachandra S., Suthipinittharm P., Luangtrakool K., Srinak D., Thongpradit R., Siriboonrit U., Tokunaga K. (2002). Association of HLA-DRB1*1502-DQB1*0501 haplotype with susceptibility to systemic lupus erythematosus in Thais. Tissue Antigens.

[110] Prinz J.C. (2018). Human Leukocyte Antigen-Class I Alleles and the Autoreactive T Cell Response in Psoriasis Pathogenesis. Front. Immunol..

[111] Noble J.A., Valdes A.M. (2011). Genetics of the HLA region in the prediction of type 1 diabetes. Curr. Diab. Rep..

[112] Bugawan T.L., Klitz W., Alejandrino M., Ching J., Panelo A., Solfelix C.M., Petrone A., Buzzetti R., Pozzilli P., Erlich H.A. (2002). The association of specific HLA class I and II alleles with type 1 diabetes among Filipinos. Tissue Antigens.

[113] Prinz J.C. (2017). Melanocytes: Target Cells of an HLA-C*06:02-Restricted Autoimmune Response in Psoriasis. J. Investig. Dermatol..

[114] Siegel R.J., Bridges S.L., Ahmed S. (2019). HLA-C: An Accomplice in Rheumatic Diseases. ACR Open Rheumatol..

[115] Kovjazin R., Carmon L. (2014). The use of signal peptide domains as vaccine candidates. Hum. Vaccin Immunother..

[116] Smith-Garvin J.E., Koretzky G.A., Jordan M.S. (2009). T cell activation. Annu. Rev. Immunol..

[117] Messaoudi I., Guevara Patiño J.A., Dyall R., LeMaoult J., Nikolich-Zugich J. (2002). Direct link between mhc polymorphism, T cell avidity, and diversity in immune defense. Science.

[118] Engels B., Engelhard V.H., Sidney J., Sette A., Binder D.C., Liu R.B., Kranz D.M., Meredith S.C., Rowley D.A., Schreiber H. (2013). Relapse or eradication of cancer is predicted by peptide-MHC affinity. Cancer Cell.

[119] Harndahl M., Rasmussen M., Roder G., Dalgaard Pedersen I., Sørensen M., Nielsen M., Buus S. (2012). Peptide-MHC class I stability is a better predictor than peptide affinity of CTL immunogenicity. Eur. J. Immunol..

[120] Vecchio F., Carré A., Korenkov D., Zhou Z., Apaolaza P., Tuomela S., Burgos-Morales O., Snowhite I., Perez-Hernandez J., Brandao B. (2024). Coxsackievirus infection induces direct pancreatic β cell killing but poor antiviral CD8^+^ T cell responses. Sci. Adv..

[121] Zheng Z., Mergaert A.M., Fahmy L.M., Bawadekar M., Holmes C.L., Ong I.M., Bridges A.J., Newton M.A., Shelef M.A. (2020). Disordered Antigens and Epitope Overlap Between Anti-Citrullinated Protein Antibodies and Rheumatoid Factor in Rheumatoid Arthritis. Arthritis Rheumatol..

[122] Mayer R.L., Verbeke R., Asselman C., Aernout I., Gul A., Eggermont D., Boucher K., Thery F., Maia T.M., Demol H. (2022). Immunopeptidomics-based design of mRNA vaccine formulations against Listeria monocytogenes. Nat. Commun..

[123] Marcu A., Bichmann L., Kuchenbecker L., Kowalewski D.J., Freudenmann L.K., Backert L., Mühlenbruch L., Szolek A., Lübke M., Wagner P. (2021). HLA Ligand Atlas: A benign reference of HLA-presented peptides to improve T-cell-based cancer immunotherapy. J. Immunother. Cancer.

[124] Ritz D., Gloger A., Weide B., Garbe C., Neri D., Fugmann T. (2016). High-sensitivity HLA class I peptidome analysis enables a precise definition of peptide motifs and the identification of peptides from cell lines and patients’ sera. Proteomics.

[125] Sarkizova S., Klaeger S., Le P.M., Li L.W., Oliveira G., Keshishian H., Hartigan C.R., Zhang W., Braun D.A., Ligon K.L. (2020). A large peptidome dataset improves HLA class I epitope prediction across most of the human population. Nat. Biotechnol..

[126] Fujiwara K., Shao Y., Niu N., Zhang T., Herbst B., Henderson M., Muth S., Zhang P., Zheng L. (2022). Direct identification of HLA class I and class II-restricted T cell epitopes in pancreatic cancer tissues by mass spectrometry. J. Hematol. Oncol..

[127] Solleder M., Guillaume P., Racle J., Michaux J., Pak H.-S., Müller M., Coukos G., Bassani-Sternberg M., Gfeller D. (2020). Mass Spectrometry Based Immunopeptidomics Leads to Robust Predictions of Phosphorylated HLA Class I Ligands. Mol. Cell. Proteom..

[128] Sudhir P.-R., Lin T.-D., Zhang Q. (2022). HLA Allele-Specific Quantitative Profiling of Type 1 Diabetic B Lymphocyte Immunopeptidome. J. Proteome Res..

[129] Caron E., Espona L., Kowalewski D.J., Schuster H., Ternette N., Alpízar A., Schittenhelm R.B., Ramarathinam S.H., Lindestam Arlehamn C.S., Chiek Koh C. (2015). An open-source computational and data resource to analyze digital maps of immunopeptidomes. eLife.

[130] Klatt M.G., Mack K.N., Bai Y., Aretz Z.E.H., Nathan L.I., Mun S.S., Dao T., Scheinberg D.A. (2020). Solving an MHC allele-specific bias in the reported immunopeptidome. JCI Insight.

[131] Bassani-Sternberg M., Chong C., Guillaume P., Solleder M., Pak H., Gannon P.O., Kandalaft L.E., Coukos G., Gfeller D. (2017). Deciphering HLA-I motifs across HLA peptidomes improves neo-antigen predictions and identifies allostery regulating HLA specificity. PLoS Comput. Biol..

[132] Shraibman B., Barnea E., Kadosh D.M., Haimovich Y., Slobodin G., Rosner I., López-Larrea C., Hilf N., Kuttruff S., Song C. (2019). Identification of Tumor Antigens Among the HLA Peptidomes of Glioblastoma Tumors and Plasma. Mol. Cell. Proteom..

[133] Yarmarkovich M., Marshall Q.F., Warrington J.M., Premaratne R., Farrel A., Groff D., Li W., di Marco M., Runbeck E., Truong H. (2023). Targeting of intracellular oncoproteins with peptide-centric CARs. Nature.

[134] Nicholas B., Bailey A., Staples K.J., Wilkinson T., Elliott T., Skipp P. (2022). Immunopeptidomic analysis of influenza A virus infected human tissues identifies internal proteins as a rich source of HLA ligands. PLoS Pathog..

[135] Parkhurst M.R., Fitzgerald E.B., Southwood S., Sette A., Rosenberg S.A., Kawakami Y. (1998). Identification of a shared HLA-A*0201-restricted T-cell epitope from the melanoma antigen tyrosinase-related protein 2 (TRP2). Cancer Res..

[136] Wang J., Jelcic I., Mühlenbruch L., Haunerdinger V., Toussaint N.C., Zhao Y., Cruciani C., Faigle W., Naghavian R., Foege M. (2020). HLA-DR15 Molecules Jointly Shape an Autoreactive T Cell Repertoire in Multiple Sclerosis. Cell.

[137] Moser J.J., Chan E.K.L., Fritzler M.J. (2013). An SNP in the trinucleotide repeat region of the TNRC6A gene maps to a major TNGW1 autoepitope in patients with autoantibodies to GW182. Adv. Exp. Med. Biol..

[138] Rosen A., Casciola-Rosen L. (2009). Autoantigens in systemic autoimmunity: Critical partner in pathogenesis. J. Intern. Med..

[139] Pedersen A.E. (2007). The potential for induction of autoimmune disease by a randomly-mutated self-antigen. Med. Hypotheses.

[140] Xiang Y., Zhang M., Jiang D., Su Q., Shi J. (2023). The role of inflammation in autoimmune disease: A therapeutic target. Front. Immunol..

[141] Muñoz L.E., Lauber K., Schiller M., Manfredi A.A., Herrmann M. (2010). The role of defective clearance of apoptotic cells in systemic autoimmunity. Nat. Rev. Rheumatol..

[142] UniProt UniProt-P54253·ATX1_HUMAN. https://www.uniprot.org/uniprotkb/P54253/entry.

[143] UniProt UniProt-O14646·CHD1_HUMAN. https://www.uniprot.org/uniprotkb/O14646/entry.

[144] UniProt UniProt-Q9UJU5·FOXD3_HUMAN. https://www.uniprot.org/uniprotkb/Q9UJU5/entry.

[145] UniProt UniProt-O14686·KMT2D_HUMAN. https://www.uniprot.org/uniprotkb/O14686/entry.

[146] UniProt UniProt-Q8NBZ0·IN80E_HUMAN. https://www.uniprot.org/uniprotkb/Q8NBZ0/entry.

[147] UniProt UniProt-Q7Z3Z5·Q7Z3Z5_HUMAN. https://www.uniprot.org/uniprotkb/Q7Z3Z5/entry.

[148] UniProt UniProt-Q6PJG2·MDEAS_HUMAN. https://www.uniprot.org/uniprotkb/Q6PJG2/entry.

[149] UniProt UniProt-Q8TBK2·SETD6_HUMAN. https://www.uniprot.org/uniprotkb/Q8TBK2/entry.

[150] UniProt UniProt-Q9Y618·NCOR2_HUMAN. https://www.uniprot.org/uniprotkb/Q9Y618/entry.

[151] UniProt UniProt-P06748·NPM_HUMAN. https://www.uniprot.org/uniprotkb/P06748/entry.

[152] UniProt UniProt-Q10571·MN1_HUMAN. https://www.uniprot.org/uniprotkb/Q10571/entry.

[153] UniProt UniProt-Q9H3D4·P63_HUMAN. https://www.uniprot.org/uniprotkb/Q9H3D4/entry.

[154] UniProt UniProt-Q15911·ZFHX3_HUMAN. https://www.uniprot.org/uniprotkb/Q15911/entry.

[155] UniProt UniProt-Q5VZL5·ZMYM4_HUMAN. https://www.uniprot.org/uniprotkb/Q5VZL5/entry.

[156] UniProt UniProt-Q96KR1·ZFR_HUMAN. https://www.uniprot.org/uniprotkb/Q96KR1/entry.

[157] UniProt UniProt-A0A2R8Y5A6·A0A2R8Y5A6_HUMAN. https://www.uniprot.org/uniprotkb/A0A2R8Y5A6/entry.

[158] UniProt UniProt-E7ENM8·E7ENM8_HUMAN. https://www.uniprot.org/uniprotkb/E7ENM8/entry.

[159] UniProt UniProt-Q6Y7W6·GGYF2_HUMAN. https://www.uniprot.org/uniprotkb/Q6Y7W6/entry.

[160] UniProt UniProt-P42858·HD_HUMAN. https://www.uniprot.org/uniprotkb/P42858/entry.

[161] UniProt UniProt-P40126·TYRP2_HUMAN. https://www.uniprot.org/uniprotkb/P40126/entry.

[162] UniProt UniProt-C9JE40·PATL2_HUMAN. https://www.uniprot.org/uniprotkb/C9JE40/entry.

[163] UniProt UniProt-A1IGU5·ARH37_HUMAN. https://www.uniprot.org/uniprotkb/A1IGU5/entry.

[164] UniProt UniProt-Q7L8J4·3BP5L_HUMAN. https://www.uniprot.org/uniprotkb/Q7L8J4/entry.

[165] UniProt UniProt-Q8NDV7·TNR6A_HUMAN. https://www.uniprot.org/uniprotkb/Q8NDV7/entry.

[166] UniProt UniProt-P51648·AL3A2_HUMAN. https://www.uniprot.org/uniprotkb/P51648/entry.

[167] UniProt UniProt-O75460·ERN1_HUMAN. https://www.uniprot.org/uniprotkb/O75460/entry.

[168] UniProt UniProt-Q9BY12·SCAPE_HUMAN. https://www.uniprot.org/uniprotkb/Q9BY12/entry.

[169] UniProt UniProt-P54098·DPOG1_HUMAN. https://www.uniprot.org/uniprotkb/P54098/entry.

[170] UniProt UniProt-P03891·NU2M_HUMAN. https://www.uniprot.org/uniprotkb/P03891/entry.

[171] UniProt UniProt-Q2M2I8·AAK1_HUMAN. https://www.uniprot.org/uniprotkb/Q2M2I8/entry.

[172] UniProt UniProt-Q5T1A1·DCST2_HUMAN. https://www.uniprot.org/uniprotkb/Q5T1A1/entry.

[173] UniProt UniProt-P21917·DRD4_HUMAN. https://www.uniprot.org/uniprotkb/P21917/entry.

[174] UniProt UniProt-Q13492·PICAL_HUMAN. https://www.uniprot.org/uniprotkb/Q13492/entry.

[175] UniProt UniProt-B7Z2A4·B7Z2A4_HUMAN. https://www.uniprot.org/uniprotkb/B7Z2A4/entry.

[176] UniProt UniProt-A0A087X0R9·A0A087X0R9_HUMAN. https://www.uniprot.org/uniprotkb/A0A087X0R9/entry.

[177] UniProt UniProt-A0A2R8Y4T1·A0A2R8Y4T1_HUMAN. https://www.uniprot.org/uniprotkb/A0A2R8Y4T1/entry.

[178] UniProt UniProt-Q9NZK5·ADA2_HUMAN. https://www.uniprot.org/uniprotkb/Q9NZK5/entry.

[179] UniProt UniProt-P0DUB6·AMY1A_HUMAN. https://www.uniprot.org/uniprotkb/P0DUB6/entry.

[180] UniProt UniProt-Q9Y264·ANGP4_HUMAN. https://www.uniprot.org/uniprotkb/Q9Y264/entry.

[181] UniProt UniProt-Q9UKU9·ANGL2_HUMAN. https://www.uniprot.org/uniprotkb/Q9UKU9/entry.

[182] UniProt UniProt-Q9Y5C1·ANGL3_HUMAN. https://www.uniprot.org/uniprotkb/Q9Y5C1/entry.

[183] UniProt UniProt-Q9BY76·ANGL4_HUMAN. https://www.uniprot.org/uniprotkb/Q9BY76/entry.

[184] UniProt UniProt-Q8NI99·ANGL6_HUMAN. https://www.uniprot.org/uniprotkb/Q8NI99/entry.

[185] UniProt UniProt-P49747·COMP_HUMAN. https://www.uniprot.org/uniprotkb/P49747/entry.

[186] UniProt UniProt-P36222·CH3L1_HUMAN. https://www.uniprot.org/uniprotkb/P36222/entry.

[187] UniProt UniProt-P02675·FIBB_HUMAN. https://www.uniprot.org/uniprotkb/P02675/entry.

[188] UniProt UniProt-P02679·FIBG_HUMAN. https://www.uniprot.org/uniprotkb/P02679/entry.

[189] UniProt UniProt-Q08830·FGL1_HUMAN. https://www.uniprot.org/uniprotkb/Q08830/entry.

[190] UniProt UniProt-O00602·FCN1_HUMAN. https://www.uniprot.org/uniprotkb/O00602/entry.

[191] UniProt UniProt-P13727·PRG2_HUMAN. https://www.uniprot.org/uniprotkb/P13727/entry.

[192] UniProt UniProt-P13611·CSPG2_HUMAN. https://www.uniprot.org/uniprotkb/P13611/entry.

[193] Malavige G.N., Jeewandara C., Ogg G.S. (2020). Dysfunctional Innate Immune Responses and Severe Dengue. Front. Cell. Infect. Microbiol..

[194] Khandia R., Munjal A., Dhama K., Karthik K., Tiwari R., Malik Y.S., Singh R.K., Chaicumpa W. (2018). Modulation of Dengue/Zika Virus Pathogenicity by Antibody-Dependent Enhancement and Strategies to Protect Against Enhancement in Zika Virus Infection. Front. Immunol..

[195] Maucourant C., Queiroz G.A.N., Samri A., Grassi M.F.R., Yssel H., Vieillard V. (2019). Zika virus in the eye of the cytokine storm. Eur. Cytokine Netw..

[196] Nanaware N., Banerjee A., Mullick Bagchi S., Bagchi P., Mukherjee A. (2021). Dengue Virus Infection: A Tale of Viral Exploitations and Host Responses. Viruses.

[197] Clé M., Desmetz C., Barthelemy J., Martin M.-F., Constant O., Maarifi G., Foulongne V., Bolloré K., Glasson Y., De Bock F. (2020). Zika Virus Infection Promotes Local Inflammation, Cell Adhesion Molecule Upregulation, and Leukocyte Recruitment at the Blood-Brain Barrier. mBio.

[198] Pavesi A., Tiecco G., Rossi L., Sforza A., Ciccarone A., Compostella F., Lovatti S., Tomasoni L.R., Castelli F., Quiros-Roldan E. (2024). Inflammatory Response Associated with West Nile Neuroinvasive Disease: A Systematic Review. Viruses.

[199] Lazarski C.A., Chaves F.A., Jenks S.A., Wu S., Richards K.A., Weaver J.M., Sant A.J. (2005). The kinetic stability of MHC class II:peptide complexes is a key parameter that dictates immunodominance. Immunity.

[200] Rasmussen M., Fenoy E., Harndahl M., Kristensen A.B., Nielsen I.K., Nielsen M., Buus S. (2016). Pan-specific prediction of peptide-MHC-I complex stability; a correlate of T cell immunogenicity. J. Immunol..

[201] George A.J.T., Stark J., Chan C. (2005). Understanding specificity and sensitivity of T-cell recognition. Trends Immunol..

[202] Fox D.A. (2015). Citrullination: A Specific Target for the Autoimmune Response in Rheumatoid Arthritis. J. Immunol..

[203] Peng Z., Xu W.W., Sham Y., Lam H., Sun D., Li C., Rasic N.F., Guan Q., James A.A., Simons F.E.R. (2016). Mosquito salivary allergen Aed a 3: Cloning, comprehensive molecular analysis, and clinical evaluation. Allergy.

[204] Conway M.J. (2021). Type I hypersensitivity promotes Aedes aegypti blood feeding. Sci. Rep..

[205] Wang Z., Liang Y., Zeng F., Li T., Cheng G. (2025). A capture enzyme-linked immunosorbent assay for detection of mosquito salivary protein-specific immunoglobulin E. PLoS Neglected Trop. Dis..

[206] Pandey R.K., Dahiya S., Mahita J., Sowdhamini R., Prajapati V.K. (2019). Vaccination and immunization strategies to design *Aedes aegypti* salivary protein based subunit vaccine tackling *Flavivirus* infection. Int. J. Biol. Macromol..

[207] Manning J.E., Morens D.M., Kamhawi S., Valenzuela J.G., Memoli M. (2018). Mosquito Saliva: The Hope for a Universal Arbovirus Vaccine?. J. Infect. Dis..

[208] Wang Y., Ling L., Jiang L., Marin-Lopez A. (2024). Research progress toward arthropod salivary protein vaccine development for vector-borne infectious diseases. PLoS Neglected Trop. Dis..

